# Human fecal transplantation from stunted children promotes metabolic dysfunction in mice fed with a high-fat and high-fructose corn syrup diet

**DOI:** 10.1080/19490976.2026.2651984

**Published:** 2026-04-02

**Authors:** Fernanda Valdez-Palomares, Lilia G. Noriega, Dana Reyes-Romo, Samuel Canizales-Quinteros, Rafael Nambo-Venegas, Citlaltepetl Salinas-Lara, Armando Tovar-Palacio, Marta Menjivar, Barbara Peña-Espinoza, Guadalupe Ortiz, Berenice Palacios-González

**Affiliations:** a Laboratorio de Envejecimiento Saludable del Instituto Nacional de Medicina Genómica en el Centro de Investigación sobre Envejecimiento, CDMX, México; b Posgrado en Ciencias Biológicas, Universidad Nacional Autónoma de México, CDMX, México; c Departamento de Fisiología de la Nutrición, Instituto Nacional de Ciencias Médicas y Nutrición Salvador Zubirán, CDMX, México; d Unidad de Genómica de Poblaciones Aplicada a la Salud, Facultad de Química, Universidad Nacional Autónoma de México/Instituto Nacional de Medicina Genómica, CDMX, México; e Laboratorio de Bioquímica de Enfermedades Crónicas, Instituto Nacional de Medicina Genómica (INMEGEN), CDMX, México; f Laboratorio de Patogénesis Molecular, Facultad de Estudios Superiores Iztacala, Universidad Nacional Autónoma de México, CDMX, México; g Instituto Nacional de Neurología y Neurocirugía Manuel Velasco Suárez, CDMX, México; h Laboratorio de Diabetes, Facultad de Química, Universidad Nacional Autónoma de México, CDMX, México; i Laboratorio de Endocrinología, Hospital Juárez de México, CDMX, México

**Keywords:** Fecal microbiota transplantation, undernutrition, obesity, malnutrition, nutritional transition, energy expenditure, visceral adipose tissue, *Akkermansia*, *Parabacteroides*

## Abstract

Stunting, or impaired child growth due to poor nutrition and infections, is characterized by a low height-for-age and affects 48%–56% of school-aged children worldwide. It is associated with later weight gain and chronic diseases. The gut microbiome in undernourished children may increase obesity risk if they are exposed to high-calorie environments. To investigate this, we assessed whether the intestinal microbiome of stunted children elevates obesity risk upon exposure to an obesogenic environment. Fecal microbiota transplantation (FMT) was performed using pooled stools from healthy (*n* = 6) or stunted (*n* = 6) school-aged children from a low-income cohort in Mexico. Eight-week-old male C57BL/6 mice underwent bowel cleansing with polyethylene glycol (PEG), followed by weekly intragastric FMT for 4 weeks. The mice were subsequently fed either a control diet (CT) or a high-fat, high-fructose corn syrup diet (HFFr, including 15% HFCS-55) for 15 weeks. Metabolic outcomes were assessed through body composition, indirect calorimetry, oral glucose tolerance test, insulin tolerance test, and histological analysis of visceral adipose tissue. The microbiota composition was evaluated by 16S rRNA V3–V4 hypervariable region sequencing, and the predicted functional capacity was analyzed using PICRUSt2. FMT from stunted children increased susceptibility to diet-induced obesity, visceral adipose tissue hypertrophy, and insulin resistance. In contrast, FMT from healthy children promoted energy expenditure and visceral adipose tissue hyperplasia, conferring a protective effect against diet-induced obesity and insulin resistance in the mice. Healthy-FMT led to sustained enrichment of *Akkermansia* and *Parabacteroides*, whereas stunting-FMT increased *Proteobacteria*, *Veillonella*, *Desulfovibrionaceae*, and *Bifidobacterium*. Microbial‒phenotypic correlations showed that *Akkermansia* and *Parabacteroides* were negatively correlated with fasting glucose, body weight, and fat mass, and positively correlated with postprandial RER, VO2, and lean mass. In conclusion, stunting-FMT recipient mice showed a higher risk of obesity and metabolic issues in an obesogenic environment. Healthy-FMT confers metabolic resilience, characterized by increased abundance of taxa such as *Akkermansia* and *Parabacteroides*, which are linked to enhanced energy expenditure, improved glucose metabolism, and favorable adipose tissue structure.

## Graphical abstract

**Figure:**
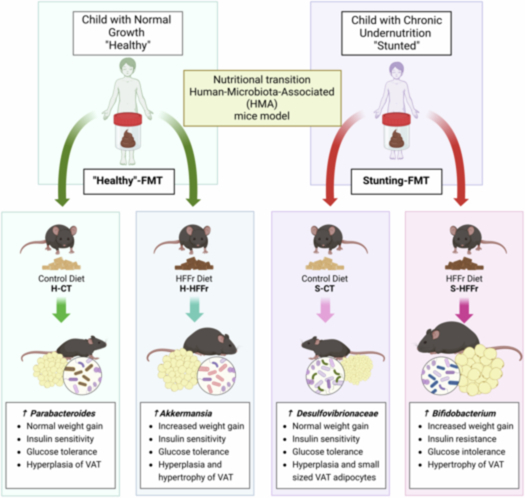

## Introduction

Undernutrition refers to inadequate provision of energy and nutrients and an inability to meet the body’s requirements to ensure growth, maintenance, and physiological functions. Globally, approximately 200 million school-aged children are undernourished.^1^ Stunting refers to chronic undernutrition, which is frequently associated with poor sanitation and poor nutrient intake.^2^ School-aged children are in an active growing stage, going through a period of rapid physical development, and are considered stunted if their height-for-age is more than two standard deviations below the WHO child growth standards median,^3^^,^^4^ serving as a visible and quantifiable physical indicator of persistent childhood malnutrition.^3^ Stunting in childhood is the most prevalent form of undernutrition globally and is estimated to affect 48%–56% of school-aged children worldwide.^5^^,^^6^ In developing countries (DC), the prevalence of stunting among school-aged children ranges from 9.3% to 24.0% in Latin America and the Caribbean, to as high as 20.2%–48.1% in Africa.^7^ Developing countries are confronting a double burden of malnutrition (DBM) due to economic growth, urbanization, and dietary patterns. This includes rising rates of obesity alongside ongoing undernutrition, often manifesting as stunting combined with overweight.^8^ Findings from the Young Lives cohort in India, Peru, and Vietnam shows that stunted individuals are more likely to be overweight, especially among lower socioeconomic groups, highlighting persistent poverty-related stunting and urban-rural disparities.^8^ Mexico exemplifies this issue, with high rates of undernutrition (29.7%) and obesity (17.6%) among school-aged children.^9-11^ Globalization and aggressive marketing have displaced traditional diets, promoting ultra-processed foods high in saturated fats and high-fructose corn syrup, which exacerbates the DBM.^12^ Biological pathways linking childhood undernutrition and increased adiposity in later life have been proposed to explain DBM and the ongoing nutritional transition.^13^ Early-life stunting is associated with impaired fat oxidation, reduced energy expenditure, and increased insulin resistance in later life.^14-17^ However, no studies have tested whether the stunting associated gut microbiome contributes to DBM in DC. Specifically, the gut microbiome facilitates the digestion of otherwise indigestible dietary components through its diverse enzymatic repertoire,^18^^,^^19^ generating short-chain fatty acids, amino acids, and vitamins.^20^ These sophisticated metabolic activities likely evolved as an adaptive strategy to maximize energy assimilation during periods of food scarcity in undernourished hosts.^21^ However, when these same microbial functions operate in obesogenic environments, they may become maladaptive, promoting excessive energy extraction and predisposing the host to obesity and metabolic dysfunction, thereby explaining the dual role of the microbiota in both undernutrition and subsequent obesity development. Supporting this paradigm, Méndez-Salazar et al. identified a chronic undernutrition-associated microbiota profile with enhanced energy harvest capacity, reflecting maladaptation to poor nutrition,^22^ which may predispose the host to obesity when dietary environments shift, connecting the pathological endpoints of malnutrition, undernutrition, and obesity. The role of the gut microbiota in obesity has been explored widely, but its involvement in the nutritional transition that occurs in DC remains underexplored.

Performing fecal microbiota transplantation (FMT) from stunted and healthy children into C57BL/6 mice, we generated a human microbiota-associated (HMA) model.^23^ Briefly, we performed bowel cleansing with polyethylene glycol (PEG) to dislodge the native gut microbiota in 8-week-old mice prior to FMT from healthy (healthy-FMT) or stunted (stunting-FMT) school-aged children. Bowel cleansing using laxative methods is an alternative approach for temporarily decreasing the abundance of gut bacteria before implantation of the donor gut microbiome.^23^^,^^24^ Evidence indicates that four consecutive bowel cleansings with PEG reduce the gut microbiota abundance by approximately 90%, achieving depletion levels comparable to those of antibiotic combinations.^23^

Chronic undernutrition alters the gut microbiota composition in ways that may enhance energy harvest.^25^^,^^26^ These microbial traits could become maladaptive when exposed to obesogenic environments, contributing to the progression from early undernutrition to later obesity. However, the causal influence of the stunting-associated microbiota on obesity susceptibility remains unclear. Transplanting the microbiota from stunted children into mice fed a high-fat and high-fructose corn syrup diet enables the evaluation of whether these microbial alterations promote diet-induced obesity and metabolic dysfunction. We hypothesized that the gut microbiome serves as a key driver of the nutritional transition from undernutrition to obesity. Building on this rationale, our research question was: “Does the gut microbiota from stunted children increase susceptibility to diet-induced obesity when transplanted into C57BL/6 mice fed a high-fat diet with HFCS-55?”.

## Materials and methods

### Collection of feces from human donors

The data and fecal samples used in the FMT experiments were obtained from a study cohort, which serves as the foundation of the current research. Briefly, fecal samples were obtained from 36 children in a cohort of 1000 school-aged children (9–11 y old) attending public schools in Chimalhuacán, México – all from low-income families.^22^ The cohort study conformed to the latest version of the Declaration of Helsinki and was approved by the Human Research Ethical Committee of Hospital Juarez de México. All parents or legal guardians and children provided written informed consent. Parents were instructed to collect the first bowel movement of the day from each child using a sterile polypropylene container. After collection, the samples were immediately transported to the laboratory facilities in ice-filled coolers and stored at −80 °C until processing.

The children were classified into three groups: stunted, normal weight, and obesity (*n* = 12 per group) based on WHO-defined criteria for height-for-age z-scores (HAZ) and BMI z-scores.^27^ Stunting was defined as HAZ ≤ −2 standard deviations (SD), obesity as BMI z-scores ≥ + 2 SD, and normal weight according to WHO BMI z-score standards. Participants were excluded from the cohort study if they had received antibiotics or been hospitalized (>24 h) in the preceding 6 months, had gastrointestinal or chronic systemic conditions, experienced diarrhea within 1 month, or used medications affecting gastrointestinal function. For fecal donor selection, we identified participants from the stunted (*n* = 6) and normal weight (*n* = 6) groups who met all of the following inclusion criteria: complete clinical, anthropometric, and biochemical data; negative parasitological tests; available microbiota sequencing data; and adequate fecal samples for FMT. The anthropometric measurements and blood serum analytes were obtained as described previously.^22^

### Fecal preparation for FMT from human donors

Only parasite-negative fecal samples from human donors were selected; they were stored at −80 °C until use for FMT into 8-week-old specific pathogen-free (SPF) mice. The fecal inocula were prepared as described previously.^23^ Briefly, the fecal samples were diluted at a 1:100 ratio in brain heart infusion (BHI, Becton Dickinson, USA) supplemented with 0.5  mg/mL L-cysteine (Sigma–Aldrich, USA), which served as a reducing medium to maintain anaerobic bacteria. To enhance the viability of bacterial stocks during storage at −80 °C, 20% skim milk (volume/volume) was added. Both the BHI and skim milk were sterilized and stored at 4 °C until use. The fecal mixture was then sequentially filtered through 2 and 1 mm meshes to remove undigested dietary residues. The resulting filtrates were separated into 200  μL aliquots and stored at −20 °C for subsequent use.

### Animals

This study utilized 8-week-old male C57BL/6 mice free from common pathogenic strains of mouse ectoparasites and endoparasites. These mice were procured from the Experimental Research Department and Animal Care Facility at the Instituto Nacional de Ciencias Médicas y Nutrición Salvador Zubirán. The mice were housed in an Optimice® (Animal Care Systems, USA) IVC rack system (2–4 animals per cage), the mice were maintained at a consistent room temperature of 24 °C (±2°C) and a 12-h light/dark cycle. During the 15-week duration of the study, the mice had free access to food and water, with weekly monitoring of their weight and food and water intake. The animal study protocols were approved by the Animal Committee of the Instituto Nacional de Ciencias Médicas y Nutrición Salvador Zubirán, Mexico City (approval no. FNU-1974).

### Bowel cleansing and human microbiota transplantation

Before transplantation, the mice were fasted for 1 h in clean cages to prevent coprophagy. This was followed by bowel cleansing, which was achieved through the intragastric administration of 200  μL of macrogol (425  g/L, CONTUMAX, Asofarma, México). This procedure was repeated four times at 20-min intervals. This PEG-mediated cleansing was performed only once during the experiment, before the initial transplant procedure in week 1. Four hours post-cleansing, the mice received 200  μL of fecal inoculum via intragastric gavage. The inoculum was sourced from healthy children (I-healthy) or stunted children (I-stunting). As a control group, we included PEG-treated mice inoculated with 0.9% NaCl instead of donor feces (PEG-only). All mice received weekly intragastric administration following a 4-h fasting period for three additional weeks (weeks 2–4); FMT groups received fecal inoculum reinforcements, and the PEG-only groups continued receiving 0.9% NaCl.

### Dietary treatments for experimental groups

Following FMT, the separate groups of mice based on the type of fecal inoculum were provided unrestricted access to distinct dietary regimens for a duration of 15 weeks. The CT diet consisted of 20.3% protein, 60.1% carbohydrates, and 19.6% lipids. The HFFr diet consisted of 20.7% protein, 27.7% carbohydrates, and 51.6% lipids and included 15% high-fructose corn syrup (HFCS-55) dispensed in sterilized water (Supplementary Table 1). These experimental diets were freshly prepared on a weekly basis, subjected to irradiation for sterilization, and stored at 4 °C before being provided to the mice in a dry form. The PEG-only groups were provided with unrestricted access to distinct dietary regimens for a duration of 5 weeks.

### Body composition analysis

Body composition was assessed biweekly using an EchoMRI™ Whole Body Composition Analyzer (EchoMRI, USA). This involved measuring the whole-body fat and lean mass of each mouse. The device was calibrated with canola oil according to the manufacturer's guidelines. For the analysis, conscious mice were gently restrained in a transparent red plastic cylinder and then placed inside the EchoMRI™ for a 2-min scan. To ensure measurement accuracy, each animal underwent duplicate scans. The total restraint time for each mouse did not exceed 10 min, and the mice were promptly returned to their cages after scanning.

### Indirect calorimetry

In the fifth week, indirect calorimetry was performed using an Oxymax Laboratory Animal Monitoring System (CLAMS, Columbus Instruments, USA). The mice were individually housed in plexiglass chambers for 24 h at a controlled room temperature of 22 °C and a 12-h light/dark cycle. Oxygen consumption (VO_2_) and CO_2_ production (VCO_2_) were recorded over two 12-h periods: one during fasting with access only to water and another with access to their respective experimental diets and water or 15% HFCS-55. The respiratory exchange ratio (RER) was calculated as the ratio of VCO_2_ to VO_2_. The results represent the average values from both the fasting and feeding periods. Linear regression analysis was employed to evaluate the changes in VO_2_ (mL/h) and EE (kcal/h) per gram of body weight, lean mass, and fat mass.

### Glucose and insulin tolerance tests

An oral glucose tolerance test (OGTT) was conducted in each mouse 13 weeks after FMT, during which time they had received their designated experimental diet. The intraperitoneal insulin tolerance test (ipITT) was performed 1 week after the OGTT. The mice were fasted for 6 h before either test. The OGTT was initiated by intragastric gavage of 2 g/kg body weight of glucose. The ipITT was initiated by the intraperitoneal administration of 0.5 U/kg body weight of insulin (Humalin® R, Eli Lilly, USA). Blood glucose levels were obtained from the tail vein using a glucometer (Abbott Diabetes Care; Freestyle Optium Neo, USA) at baseline and 15, 30, 45, 60, 90, and 120 min after the administration of glucose for the OGTT, and at baseline and 20, 40, 60, 90, and 120 min after the administration of insulin for the ipITT.

### Histological analysis

VAT was fixed in 4% paraformaldehyde medium and embedded in paraffin. Two consecutive sections (4 μm) per mouse were cut from each tissue and stained with hematoxylin and eosin. Five images were randomly collected from each section per mouse for analysis. Images were acquired using a Leica ESTELLARIS 5 confocal microscope with a 10 ×  objective. The adipocyte area and the number of adipocytes per field were quantified using FIJI image software and the Adiposoft plugin.^28^

### Fecal sampling of FMT-recipient mice

Fecal pellets were aseptically collected from plastic cages (*n* = 6 pooled samples) at week 0 (baseline). At week 5 (1-week post-FMT), the fecal pellets were collected individually (*n* = 3–8 per group). At week 12, the fecal pellets were collected from plastic cages (*n* = 2–3 pooled samples per group, each pool representing 2–3 mice). All the collected fecal pellets were immediately snap-frozen and stored at −80 °C until DNA extraction.

### DNA isolation, sequencing, and bioinformatics analysis

DNA from human donors’ fecal inocula and fecal samples from FMT- recipient mice was isolated using the QIAGEN Power Fecal pro kit according to the manufacturer's instructions (Qiagen, Hilden, NRW, Germany). Purified DNA samples were verified qualitatively for integrity by agarose gel electrophoresis, and the concentration was estimated using a Nanodrop 8000 (Thermo Fisher Scientific, Hudson, NH, USA). The 16S rRNA V_3_–V_4_ hypervariable region was amplified using the Bakt_341F and Bakt_805R primers.^29^ Each PCR reaction was carried out in a final volume of 50 µL comprising 25 µL of 1x Platinum Multiplex PCR Master Mix (Applied Biosystems, Massachusetts, USA), 100 nM of each forward and reverse primer, and 100 ng of bacterial DNA on a Thermo ABI GeneAmp 9800 Fast Thermal Cycler (Applied Biosystems, Massachusetts, USA). The thermal cycling conditions were optimized as 95 °C for 4 min, followed by 25 cycles of 95 °C for 30 s, 55 °C for 30 s, and 72 °C for 3 min. PCR products were verified qualitatively for integrity by agarose gel electrophoresis, and the concentrations were estimated using a Nanodrop 8000. DNA libraries were sequenced at the Sequencing Unit of the Instituto Nacional de Medicina Genómica using MiSeq Illumina (Illumina, San Diego, CA, USA) with the MiSeq Reagent v3 Kit.

The 16S rRNA V_3_–V_4_ sequencing data and metadata of fecal donors were obtained from the Méndez-Salazar Chimalhuacán pediatric study cohort.^22^ Raw multiplexed paired-end Illumina FASTQ reads from human donors, fecal inocula for FMT and fecal samples from FMT-recipient mice were processed using Quantitative Insights into Microbial Ecology 2 (QIIME2) pipeline.^30^

Raw multiplexed paired-end Illumina FASTQ reads were trimmed for adapters and barcodes using the cutadapt q2-plugin. Quality filtering, chimera removal, and dereplication of demultiplexed reads were performed using the DADA2^31^ denoising algorithm via the q2-dada2 plugin. Taxonomic assignment of the resulting amplicon sequence variants (ASVs) was performed using the q2-feature-classifier plugin with a machine-learning naïve Bayes classifier. Two classifiers were trained: one targeting the 319F/806R region to annotate fecal donor reads based on the Méndez-Salazar sequencing pipeline, and a second targeting the Bakt_341F/Bakt_805R region to annotate human fecal inocula and FMT-recipient mice reads. Both classifiers were trained using the SILVA 132 database (99% OTUs, full-length sequences). QIIME2-processed data were imported into R (version 4.1.3) using qiime2R (version 0.99.6) and Phyloseq ^32^ (version 1.52.0) packages for taxonomy visualization and data analysis.

Differentially abundant taxa in fecal donors according to nutritional phenotype were identified using Linear Discriminant Analysis Effect Size (LEfSe) implemented in the MicrobiomeMarker (version 1.0.2).^33^^,^^34^ Features with LDA scores > 2 and *p*-values < 0.05 were considered statistically significant. A rooted phylogenetic tree was generated with VEGAN (version 2.7.2) to compute diversity metrics in FMT-recipient mice. Alpha diversity (Chao1, Shannon, and Simpson indices) and beta diversity (Bray–Curtis distances) were calculated using phyloseq (version 3.22).^32^ Differences in alpha diversity among groups were evaluated using analysis of variance (ANOVA) or the Kruskal–Wallis test, as appropriate based on data distribution. Permutational multivariate analysis of variance (PERMANOVA, adonis2) was performed using VEGAN^35^ to compare the microbial community structures among the experimental groups. Principal coordinate analysis (PCoA) and hierarchical clustering, which are based on Bray–Curtis distances and Ward’s method, were generated using the microviz (version 0.12.7)^36^ and dendextend (version 1.19.1)^37^ packages, respectively. The presence of fecal inoculum-derived ASVs in recipient mice after FMT was evaluated using ggVenn (version 1.1.10). Effect sizes of differentially abundant taxa were further estimated using LEfSe implemented in MicrobiomeMarker (version 1.0.2), applying a *p*-value < 0.05 and an LDA threshold > 2.

Correlations between bacterial genera and physiological variables obtained from FMT-recipient mice were assessed using Spearman’s rank correlation with the Hmisc package (version 5.0-1). *P*-values < 0.05 were considered statistically significant, and correlations were visualized using the pheatmap package (version 1.0.12). Rows (bacterial genera) and columns (physiological variables) were ordered by hierarchical clustering based on Euclidean distances.

Network analysis of linear relationships at the genus level, comparing FMT recipient mice, was performed using Sparse Estimation of Correlations among Microbiomes (SECOM)^38^ in the MicrobiomeAnalyst platform.^39^ Functional metagenomic predictions were inferred using PICRUSt2.^40^ Unstratified MetaCyc pathway abundances were predicted using default parameters. Differential abundance analysis of the KEGG Orthology (KO), Enzyme Commission (EC), and MetaCyc pathways by ALDEx2 was carried out using the ggpicrust2 package (version 1.7.1).

### Statistical analysis

Data obtained from the metabolic phenotypes of the mice were analyzed using GraphPad Prism 9.0 software. The results were assessed by one-way ANOVA and Tukey's post-hoc test to identify significant differences among experimental groups unless otherwise stated in the figure legends. The results are presented as mean ±  standard error. Differences between means were compared at a level of significance of *P* < 0.05.

## Results

### Anthropometric and biochemical characteristics of fecal donors

To classify fecal donors according to their nutritional status, we evaluated children according to the HAZ. This classification yielded normal linear growth (i.e., healthy; *n* = 6) and impaired growth (i.e., stunted; *n* = 6) groups with a mean cohort age of 10 y. Comprehensive nutritional phenotyping revealed clinically significant deficits in the stunted group, demonstrating severe growth retardation (mean HAZ −2.35 ± 0.27) without wasting (BMI Z-score −1.06 ± 0.89) or reduced bone mass percentage, supporting the diagnosis of chronic undernutrition (Table 1). Biochemical and hormonal serum markers were within the normal range according to age in both groups of fecal donors, but significant differences were observed in the serum concentrations of glucose, total protein, and alanine aminotransferase (Table 2). These findings highlight metabolic and nutritional disparities among the donors, establishing a baseline for evaluating the effects of donor nutritional status in subsequent analyses.

**Table 1.** Anthropometric characteristics of fecal donors.

	Healthy (*n* = 6)	Stunting (*n* = 6)
Age (years)	10.33 ± 0.52	10.33 ± 1.21
Sex	4 boys/2 girls	1 boy/5 girls
Height (cm)	142.63 ± 6.95	135.53 ± 9.38
Weight (kg)	35.55 ± 4.2	28.32 ± 4.31
BMI (kg/m^2^)	17.32 ± 0.72	15.40 ± 1.50*
BMI-for-age (Z-score)	0.13 ± 0.38	−1.06 ± 0.89*
Height-for-age (Z-score)	−0.38 ± 0.52	−2.35 ± 0.27***
Tricipital skinfold (mm)	12.17 ± 4.02	9.33 ± 4.23
Tricipital skinfold percentile	37.83 ± 20.20	2.33 ± 18.68*
Fat mass (%)	12.22 ± 3.42	10.86 ± 3.01
Bone mass (%)	1.67 ± 0.16	1.33 ± 0.20**

Two-tailed Welch test for unrelated samples. Significance values:

^*^ *p* < 0.05.

^**^ *p* < 0.001.

^**^ *p* < 0.0001.

**Table 2.** Serum biochemical markers of fecal donors.

	Healthy (*n* = 6)	Stunting (*n* = 6)
Glucose (mmol/L)	4.64 ± 0.26	4.37 ± 0.11*
Cholesterol (mmol/L)	3.34 ± 0.31	3.26 ± 0.24
VLDL (mmol/L)	0.31 ± 0.01	0.33 ± 0.07
LDL (mmol/L)	2.20 ± 0.59	2.38 ± 0.34
HDL (mmol/L)	1.39 ± 0.17	1.17 ± 0.18
Triglycerides (mmol/L)	0.66 ± 0.14	0.71 ± 0.16
Urea nitrogen (mmol/L)	2.89 ± 0.67	3.32 ± 0.67
Uric acid (mmol/L)	0.23 ± 0.05	0.26 ± 0.02
Urea (mmol/L)	2.91 ± 0.68	3.29 ± 0.69
Total protein (g/dL)	7.2 ± 0.3	7.6 ± 0.1*
Albumin (mmol/L)	0.05 ± 0.00	0.06 ± 0.00
AST (U/L)	30.8 ± 3.9	31.0 ± 3.0
ALT (U/L)	16.8 5.7	10.1 ± 2.4*
Insulin (µU/mL)	4.5 ± 1.8	3.8 ± 1.6
Leptin (µU/mL)	159.3 ± 131.3	223.9 ± 99.3
Adiponectin (µU/mL)	111.7 ± 35.8	124.4 ± 45.8
HOMA-IR	0.9 ± 0.40	0.75 ± 0.30

Two-tailed Welch test for unrelated samples. Significance values:

^*^ *p* < 0.05.

**p < 0.001.

*** *p* < 0.0001.

### Taxonomic analysis of fecal donors' samples

**Figure 1.**
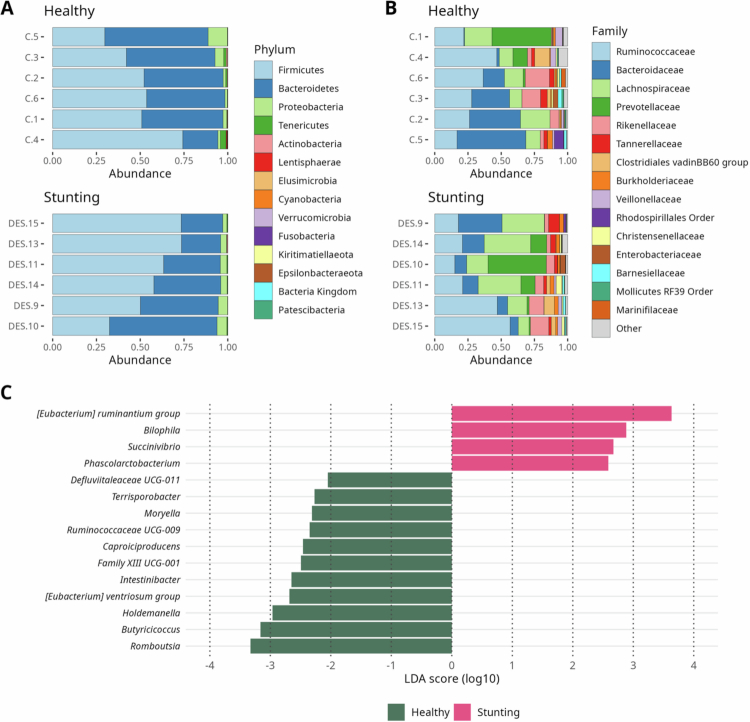
Taxonomic composition of the intestinal microbiota in Healthy and Stunted donors. (A, B) Phylum- and family-level taxonomic resolution. (C) Linear discriminant analysis of effect size (LDA-LefSe) at the genus level. LDA score threshold < 0.2 was set and *P*-value < 0.05 was considered statistically significant.

The predominant phyla in both groups of donors were Firmicutes, Bacteroidetes, Proteobacteria, Tenericutes, and Actinobacteria (Figure 1A). The taxa with the highest relative abundance at the family level were Ruminococcaceae, Bacteroidaceae, Lachnospiraceae, Prevotellaceae, Rikenellaceae, Tannerellaceae, and Clostridiales vadin BB60 (Figure 1B). To investigate the presence of microorganisms and explain the differences between the microbial communities of the healthy and stunted groups, LEfSe used an alpha value of 0.05 and a logarithmic threshold of 2. The following genera were identified in the stunted group: *Eubacterium ruminantium group*, *Bilophila, Succinivibrio,* and *Phascolarctobacterium*. In the healthy group, *Romboutsia*, *Butyricicoccus*, *Holdemanella*, *Eubacterium ventrosium group*, *Intestinibacter*, *Family XIII UCG-001*, *Caproiciproducens*, *Ruminococcaceae UCG-009*, *Moryella*, *Terrisporobacter,* and *Defluvitaleaceae UCG-001* were identified (Figure 1C). As the stunted donors were mainly female, we analyzed the microbial diversity to eliminate sex-driven differences in fecal donors (Supplementary Figure 1 A, B). No differences in Bray‒Curtis or weighted UniFrac *β*-diversity were observed when comparing fecal donors by sex.

### Bacterial engraftment dynamics in recipient mice following FMT

**Figure 2.**
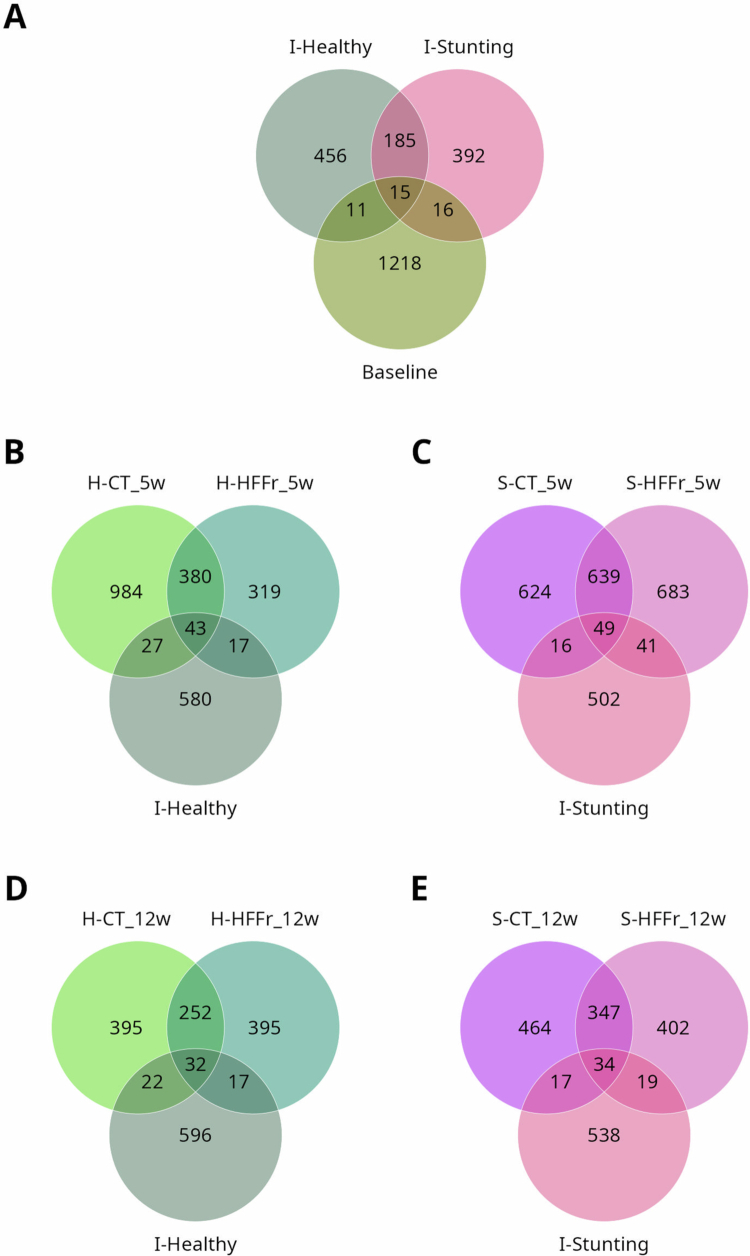
Short- and long-term persistence of transplanted ASVs in recipient mice. Venn diagrams of (A) ASVs shared between I-healthy and I-stunting fecal inocula and mice at baseline. (B) ASVs shared between I-healthy and healthy-FMT recipients, (C) between I-stunting and stunting-FMT recipients at week 5, (D, E) and week 12.

To determine whether the intestinal microbiome of chronically undernourished children increases the risk of developing obesity upon exposure to an obesogenic environment, we employed an HMA model^18^ using bowel cleansing with PEG to dislodge the native gut microbiota of C57BL6 mice prior to healthy-FMT or stunting-FMT compared to the PEG-only control group. The mice were then fed a CT diet or an HFFr diet. To confirm bacterial engraftment of the donor’s microbiota, we tracked 1075 inoculum-derived ASVs in recipient mice after FMT (Figure 2). ASV analysis of inocula revealed 667 ASVs in I-Healthy and 608 in I-Stunting (200 shared), whereas recipient mice harbored 1260 ASVs at baseline (before FMT) with limited donor overlap (11–16 ASVs; Figure 2A). At week 5, the healthy-FMT group fed the CT diet (H-CT) retained 70 ASVs, the healthy-FMT group fed the HFFr diet (H-HFFr) retained 60 ASVs, the stunting-FMT group fed the CT diet (S-CT) retained 65 ASVs, and the stunting-FMT group fed the HFFr diet (S-HFFr) retained 90 ASVs (Figure 2B, C). By week 12, persistence declined to 54 (H-CT), 49 (H-HFFr), 51 (S-CT), and 53 (S-HFFr) ASVs, with donor-specific signatures. The mice in the healthy-FMT group were characterized by engraftment of the genera *Lactobacillus* and *Bilophila,* whereas the mice in the stunting-FMT group were characterized by *UBA1819*, *Veillonella,* and *Holdemania* (Figure 2C, D). Diet-modulated engraftment was also observed; *Faecalibacterium* was prominent in the CT diet-fed mice, whereas *Enterococcus*-belonging ASVs were mainly observed in the mice on the HFFr diet (Supplementary File 1).

The impact of PEG on bacterial dynamics was also assessed. Analysis revealed the presence of 93 ASVs unique to mice in the PEG-only group, potentially reflecting a post-cleansing bloom of opportunistic taxa, low-abundance bacteria at baseline unmasked by PEG-mediated depletion of dominant taxa, or even environmental bacteria introduced during the protocol. A total of 196 ASVs were shared by all FMT recipients and PEG-only mice, with the healthy-FMT group having a stronger overlap with the PEG-only group (63 ASVs; *Lachnospiraceae, Lactobacillus,* and *Faecalibacterium*) than the stunting-FMT group (33 ASVs; *Ruminiclostridium*, *Streptococcus,* and *Ureaplasma*). A total of 294 baseline ASVs were resistant to both PEG and FMT (*Mucispirillum schaedleri ASF457, Akkermansia*, *Lactobacillus, Lactobacillus, Helicobacter*, *Desulfovibrio* species, *Streptococcus, Ruminiclostridium 5, Ruminiclostridium 9, Bacteroides acidifaciens, Bacteroides massiliensis, Alistipes,* and *Odoribacter*). This is particularly relevant because this group of PEG-resistant bacteria represents a core of stress-resistant bacterial colonizers that could determine the successive engraftment dynamics of taxa according to donor‒diet interactions (Supplementary Figure 2A). The healthy-FMT group uniquely retained 189 ASVs (*Faecalibacterium prausnitzii, Parabacteroides johnsonii,* and *Holdemania massiliensis*) versus 121 ASVs in the stunting-FMT group (*Enterococcus, Desulfovibrio, Ruminiclostridium,* and *Mucispirillum*), with differential baseline ASV retention. ASVs belonging to the genus *Akkermansia* were present in both I-healthy and I-stunting and in recipient mice before and after FMT, regardless of whether the mice were fed the CT or HFFr diet, reflecting the robust adaptability of this taxa to proliferate in the guts of different species despite the consumption of different diets. ASVs detected across groups are depicted in Supplementary File 2. This analysis highlights the complex interplay of the donor microbiota, PEG conditioning, and diet in shaping bacterial ecology during engraftment.

### β-diversity of FMT recipient mice was persistently modified according to the nutritional status of the fecal donor

**Figure 3.**
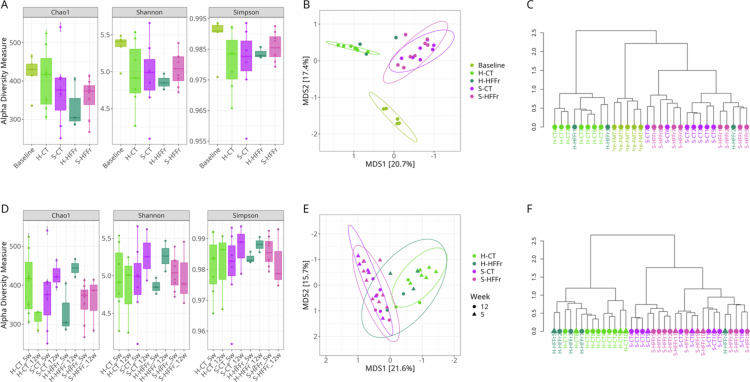
The nutritional status of FMT donors modified bacterial diversity in recipient mice. The effect of FMT on bacterial diversity in experimental groups was determined using alpha diversity metrics (richness: Chao1; evenness: Shannon and Simpson indices), and beta diversity was evaluated using Bray–Curtis dissimilarity distances. (A) Alpha diversity indices. (B) Bray–Curtis-based principal coordinate analysis (PCoA). (C) Bray–Curtis-based hierarchical clustering comparing mice at baseline and experimental groups at week 5. (D) Alpha diversity indices. (E) Bray–Curtis-based principal coordinate analysis. (F) Bray–Curtis-based hierarchical clustering comparing experimental groups of mice at weeks 5 and 12 to assess the long-term effects of FMT on bacterial diversity in recipient mice. Pairwise Wilcoxon rank-sum tests were conducted for alpha diversity, with no significant differences observed. PERMANOVA was carried out to assess beta diversity; *P* < 0.05 was considered statistically significant. Fecal pellets were collected at week 0 (baseline: *n *= 6, pooled samples), week 5 (one week post-FMT: *n *= 3–7 individual samples per group), and week 12 (*n *= 2–3 pooled samples per group, each pool representing 2–3 mice).

To evaluate the sustained influence of FMT, we analyzed *α*- and *β*-diversity in recipient mice before and after transplantation. At week 5 (one-week post-FMT), no significant differences in *α*-diversity were observed relative to baseline (pre-FMT) (Figure 3A, Supplementary Tables 2–4). In contrast, Bray–Curtis *β*-diversity analyses (PCoA and hierarchical clustering) showed clear separation between the healthy-FMT and stunting-FMT groups, driven primarily by donor nutritional status rather than by the recipient diet (Supplementary Table 5; Figure 3B, C). This donor-dependent clustering persisted through week 12 (Supplementary Table 6; Figure 3E, F), indicating a durable restructuring of the gut microbiota following FMT. The PEG-only controls (PEG-CT and PEG-HFFr) overlapped with the baseline and healthy-FMT samples at week 5 (Supplementary Table 7; Supplementary Figure 2B), suggesting that PEG cleansing transiently perturbed the gut microbiota. To quantify the relative contributions of PEG and the donor microbiota, we performed *β*-diversity analyses restricted to the baseline, PEG-only, and healthy-FMT groups (Supplementary Table 8). Both factors significantly explained the variation in community structure (healthy-FMT donor: R² = 0.13, *p* = 0.001; PEG: R² = 0.18, *p* = 0.001), confirming that PEG exerts a measurable but distinct effect from FMT. A similar pattern was observed for stunting-FMT recipients (Supplementary Table 9), which clustered separately from PEG-only controls (stunting-FMT donors: R² = 0.14, *p* = 0.001; PEG: R² = 0.12, *p* = 0.001), demonstrating again that PEG and donor origin contributed independently to *β*-diversity shifts.

Together, these findings show that PEG cleansing and FMT produced separable effects on the gut microbial composition. Although PEG induced a short-lived disturbance that may facilitate initial engraftment, it did not reproduce the *β*-diversity patterns characteristic of either donor microbiota. The persistent donor-specific clustering from weeks 5 to 12 – despite identical dietary conditions in recipients – demonstrates that FMT, rather than PEG or diet, was the primary driver of long-term microbiota restructuring.

### Healthy-FMT increased *Akkermansia* and *Parabacteroides* abundance in recipient mice

**Figure 4.**
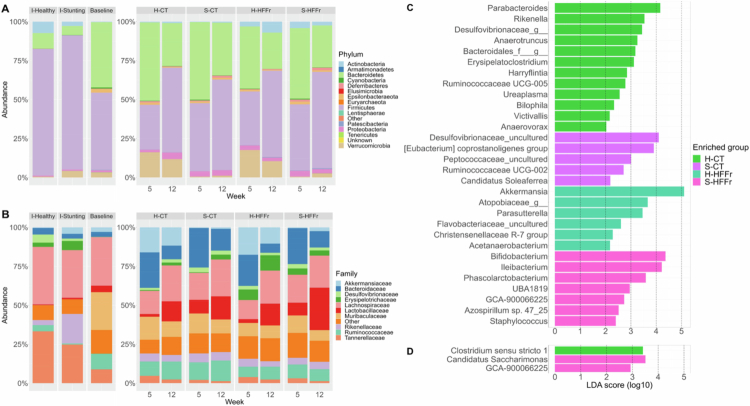
Effect of FMT and diet on the microbial abundance of recipient mice. (A, B) Phylum- and family-level taxonomic resolution: The left panel of the figures corresponds to mice at baseline and fecal inocula. The right panel of the figures corresponds to transplanted mice at weeks 5 and 12, across the experimental groups, reflecting long- and short-term effect of FMT and diet on bacterial composition. (C, D) Differentially abundant features identified using LEfSe algorithm among the experimental groups at weeks 5 and 12, respectively, the LDA score threshold < 0.2 was set, and *P*-value < 0.05 was considered statistically significant.

Healthy-FMT had a remarkable influence on the abundance of specific microbial taxa in recipient mice, indicating a sustained impact on microbiota composition. We analyzed the microbiota profile at the phylum and family levels, highlighting the intricate dynamics shaped by FMT and dietary conditions. Both fecal inocula exhibited dominance of phyla Firmicutes, Bacteroides, and Verrucomicrobia. Prominent families included Lachnospiraceae, Akkermansiaceae, Peptostreptococcaceae, Tanerellaceae, and Clostridiaceae_1. Prior to FMT, recipient mice were dominated by Firmicutes, Bacteroides, Verrucomicrobia, Proteobacteria, and Epsilonbacteraeota, with key families including Lachnospiraceae, Muribaculaceae, Lactobacillaceae, Ruminococcaceae, and Helicobacteriaceae (Figure 4A, B). FMT induced changes in the composition of the microbiota in recipient mice. Mice in the Stunting-FMT group had an increase in Proteobacteria and Lactobacillaceae and decrease in Bacteroidaceae, whereas mice in the Healthy-FMT group had a decrease in Firmicutes and increase in Verrucomicrobia and Akkermansiaceae (Figure 4A, B).

Notably, the composition of the microbiota of the recipient mice did not return to the pre-FMT composition during the study period, not even in week 12. In addition, the HFFr diet induced enrichment of Actinobacteria in all FMT groups compared to the CT diet. Differential abundance analysis at week 5 at the genus level revealed that *Parabacteroides* and *Akkermansia* enrichment in H-CT and H-HFFr, respectively, whereas S-CT and S-HFFr showed enrichment of *Desulfovibrionaceae_uncultured* and *Bifidobacterium*, respectively (Figure 4C). The number of differentially abundant taxa among groups decreased by week 12, emphasizing the time-dependent nature of interspecies FMT (Figure 4D).

To distinguish true donor-derived effects from PEG-induced artifacts, we compared baseline, PEG-only, healthy-FMT, and stunting-FMT mice (Supplementary Figure 3). *Rikenella* and *Anaerotruncus* – initially identified as differentially abundant in the H-CT group – were also enriched in PEG-only mice, demonstrating that their variability was attributable to PEG cleansing rather than to FMT. In contrast, *Akkermansia* and *Parabacteroides* were consistently modulated only in healthy-FMT recipients, and no PEG-associated taxa were detected in stunting-FMT mice. Together with *β*-diversity analyses, these results demonstrate that PEG cleansing and FMT generate separable and quantifiable effects on the gut microbial structure. PEG introduced taxonomic shifts that were distinguishable from those associated with donor-derived modulation, whereas healthy-FMT exerted donor-specific reshaping of *Akkermansia* and *Parabacteroides*. In contrast, the microbiota of stunting-FMT recipients remained largely unresponsive to PEG-induced, suggesting an intrinsic resilience or dysfunction limiting its capacity to be remodeled. Overall, these findings underscore that long-term microbiota restructuring was driven primarily by the nutritional phenotype of the donor rather than by PEG cleansing or diet.

### Microbial interaction dynamics in FMT recipient mice and the *Akkermansia*–*Parabacteroides* axis

**Figure 5.**
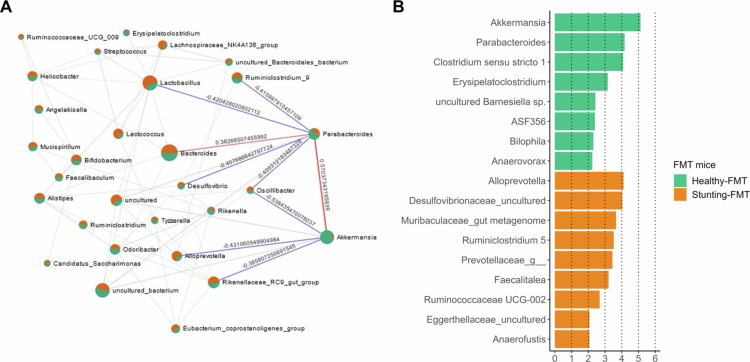
Microbial interaction networks in FMT recipient mice. (A) The interaction network using SECOM correlation coefficients at the genus level in the fecal microbiota of healthy-FMT and stunting-FMT recipient mice. The nodes represent genera; the edges illustrate the correlation coefficients. The blue lines indicate negative correlation and the red lines indicate positive correlations with *Akkermansia* and *Parabacteroides* genus. Nodes are colored according to their abundance among groups. (B) Global effect on differentially abundant features identified using LEfSe algorithm comparing healthy-FMT and stunting-FMT recipient mice. The LDA score threshold < 0.2 was set, and the *P*-value < 0.05 was considered statistically significant.

To investigate the presence of bacterial interaction networks according to the nutritional status of the fecal donor, which may potentially modulate the composition of the microbiota of recipient mice after FMT, SECOM analysis was conducted for bacterial genera within the microbiota of the HMA model. We identified microbial players with direct interactions with *Akkermansia*, including *Rikenellaceae_RC9_gut_group*, *Alloprevotella*, and *Oscillibacter*, which displayed negative associations with *Akkermansia*. This suggests a potential inhibitory role, hindering the proliferation of *Akkermansia*. Notably, the *Parabacteroides* genus exhibited a positive correlation with *Akkermansia* (Figure 5A). Furthermore, enrichment analysis revealed higher abundance of both *Akkermansia* and *Parabacteroides* in the healthy-FMT group compared to the stunting-FMT group (Figure 5B). This enrichment pattern suggests a potential cooperative interaction between *Akkermansia* and *Parabacteroides*, contributing to the observed differences based on the nutritional status of the fecal donor. The positive correlation between *Akkermansia* and *Parabacteroides* suggests a mutualistic relationship, where the presence and activities of these genera may be interdependent, influencing the overall microbial landscape. Consequently, microbial networks potentially impact FMT outcome and success and subsequent alterations in the microbiota of recipient mice. The differential enrichment observed in mice in the healthy-FMT vs. stunting-FMT groups indicates that the nutritional status of the fecal donor plays a crucial role in shaping the composition of the microbiota of recipient mice. This analysis revealed a directed microbial interactome, highlighting the potential inhibitory and mutualistic relationships surrounding *Akkermansia*, with implications for comprehending the intricate dynamics of FMT outcomes.

### Reduced weight gain in healthy-FMT recipient mice compared to stunting-FMT recipient mice when fed the HFFr diet

**Figure 6.**
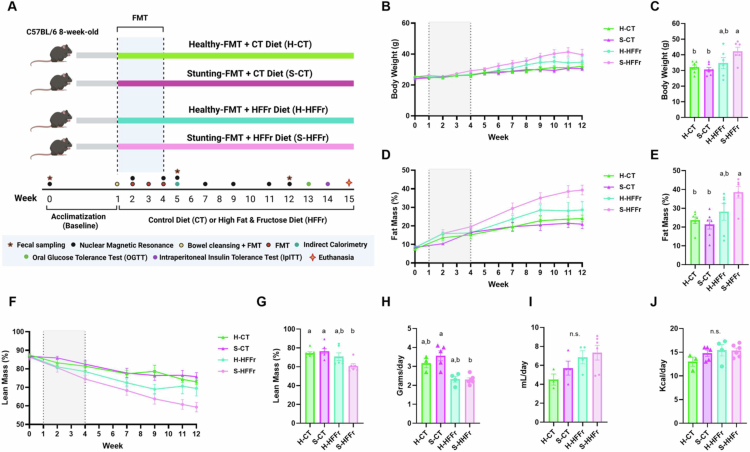
Stunting-FMT and HFFr diet effects on metabolic parameters in humanized mice. (A) Experimental model design: after a 1-week acclimatization period, 8-week-old C57BL6 male mice underwent FMT. At week 1, after bowel cleansing, the mice received either healthy-FMT + Control diet (H-CT, *n* = 7) or high-fat & fructose diet (H-HFFr, *n* = 6), or stunting-FMT + Control diet (S-CT, *n* = 7) or high-fat & fructose diet (S-HFFr, *n* = 8). FMT was performed weekly until week 4. Metabolic tests (indirect calorimetry, OGTT, and IpITT) and body composition analysis (NMR) were conducted post-FMT. Fecal samples were collected at week 0 (pooled, *n* = 5), week 5 (individual), and week 12 (pooled). (B, C) Body weight gain (trajectory curve and week 12  bar graph). (D, E) Fat mass percentage (FM%) (trajectory curve and week 12  bar graph). (F, G) Lean mass percentage (LM%, trajectory curve and week 12 bar graph). (H–J) Daily intake of diet in grams, daily intake of water and energy per mouse. The data are presented as mean ± SEM. Mixed-effect analysis was performed; different letters (a > b) indicate significant differences (*p* < 0.05). In trajectory plots (B, D, F), gray boxes denote the FMT treatment period (weeks 1–4).

To determine whether stunting-FMT promotes long-term obesity, we analyzed body weight gain and composition in recipient mice throughout the study (Figure 6A). At week 12, the S-CT and H-CT mice had the lowest body weight and fat mass percentage (FM%) among the groups. Conversely, the S-HFFr mice had the highest body weight and FM% and the lowest lean mass percentage (LM%) compared with H-CT and S-CT mice. Mice fed the CT diet only had lower body weight and FM% when compared to S-HFFr mice; differences were no longer observed when compared with H-HFFr mice (Figure 6B–G). These results indicate that exposure to an obesogenic diet resulted in increased body weight and fat mass and decreased lean mass when the fecal donor was undernourished. S-CT mice had a higher daily dietary intake in grams per mouse compared to S-HFFr mice; no differences were observed among the other groups. Similarly, no differences were found in daily water or energy intake among the groups of recipient mice (Figure 6H–J).

### Healthy-FMT led to lower glycemia and higher oxygen consumption and energy expenditure than in S-HFFr mice

**Figure 7.**
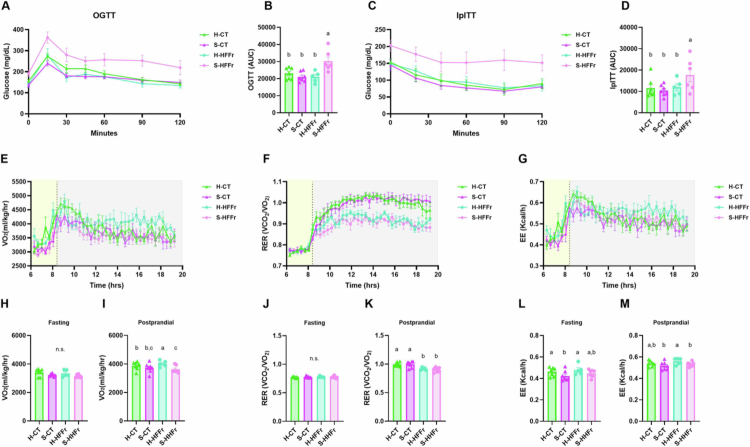
Healthy FMT led to lower glycemia and higher oxygen consumption in mice fed the HFFr diet. (A) OGTTs were conducted on mice at week 13 (post-TMF). (B) Glucose AUC during the OGTT. (C) IpITT was conducted on mice at week 14 (post-FMT). (D) Glucose AUC during the IpITT. Indirect calorimetry was conducted at week 5 (post-FMT). (E–G) VO_2_ consumption, RER and EE trajectories during the experiment. Fasting and postprandial (H, I) VO_2_ consumption (ml/kg/hr), (J, K) RER (VCO_2_/VO_2_) and (L, M) EE (kcal/h). The results are shown as the mean ± S.E.M. Two-way ANOVA was conducted, followed by Tukey’s post-hoc test. Different letters indicate a significant difference (a > b) *P* < 0.05. In figures A and D, yellow and gray boxes indicate the fasting and postprandial periods, respectively.

To evaluate whether FMT affects long-term glucose tolerance and insulin sensitivity, OGTT, and ipITT were conducted at weeks 13 and 14, respectively. S-HFFr mice had the highest area under the curve (AUC) during both the OGTT (Figure 7A, B) and the ipITT (Figure 7C, D). Notably, the trajectories and AUC of H-HFFr mice during the OGTT and ipITT highly resembled those of mice from either FMT group fed the CT diet. These results indicate that the glucose alterations observed in the mice were dependent on the interaction of diet and donor microbiota, as healthy-FMT recipient mice presented better glucose tolerance than stunting-FMT recipient mice when exposed to an obesogenic diet.

To determine whether the nutritional status of the FMT donor could modify EE and metabolic substrate use, indirect calorimetry was performed in the recipient mice at week 5 (Figure 7E–G). VO_2_ (mL/kg/h) and VCO_2_ were determined (Figure 5E). No differences in fasting VO_2_ were observed among the groups (Figure 7H). However, the S-HFFr mice had lower postprandial VO_2_ compared to the healthy-FMT groups (Figure 7I). Interestingly, H-HFFr mice had the highest postprandial VO_2_ relative to all the other groups, and H-CT mice had higher VO_2_ values than S-HFFr mice. Subsequently, substrate utilization was determined by the RER. We did not find any differences in the fasting RER among the groups (Figure 7J). However, the postprandial RER was higher in CT diet-fed mice compared to HFFr diet-fed mice (Figure 7K). No differences in the RER were observed according to FMT donor, indicating that diet determined the postprandial utilization of substrates. EE analysis of FMT recipients showed that healthy-FMT recipient mice had higher EE than S-CT mice in both the fasting and postprandial states. When fed, H-HFFr mice exhibited the highest EE, whereas stunting-FMT recipient mice had significantly lower EE (Figure 7L, M).

**Figure 8.**
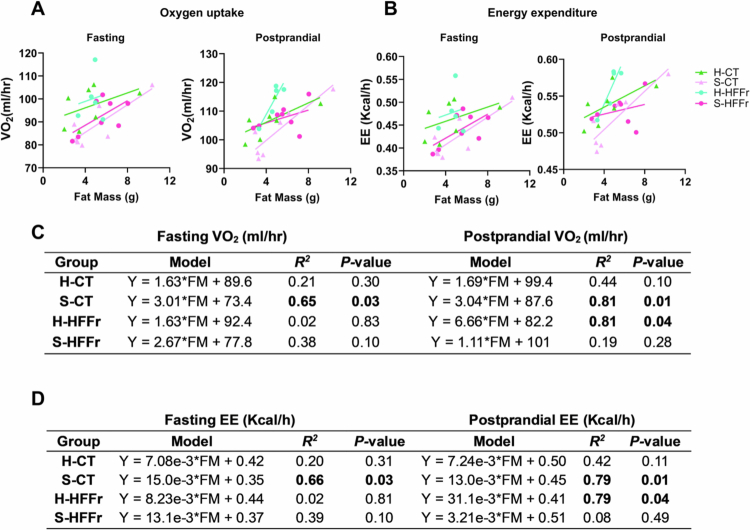
Linear regression analysis of energy expenditure and oxygen consumption in FMT-recipient mice. The effect of FMT on (A) oxygen consumption and (B) energy expenditure in mice were evaluated by linear regression analysis, with oxygen consumption during the fasting and postprandial periods according to grams of fat mass. (C, D) Linear regression equations for each group (*n* = 5–8 by group).

To explore whether the FMT donor's nutritional status modified metabolism based on body composition, linear regression analysis was performed to predict VO_2_ (mL/h) and EE (kcal/h) per gram of body weight, fat mass, and lean mass. H-HFFr mice exhibited the highest fasting and postprandial VO_2_ and EE per gram of body weight (Supplementary Figure 4A–F). Similarly, the highest postprandial VO_2_ and EE per gram of fat mass were observed in H-HFFr mice (Figure 8A–D). H-HFFr mice also demonstrated the highest fasting and postprandial VO_2_ and EE per gram of lean mass (Supplementary Figure 4G-L). These findings collectively suggest that healthy-FMT promoted increased VO_2_ and EE in recipient mice despite consumption of the HFFr diet, implying that differentially engrafted taxa may contribute to this metabolic response.

Taken together, the OGTT, ipITT, and indirect calorimetry results provide an integrated view of the metabolic phenotype. The improved glucose tolerance and insulin sensitivity in the H-HFFr group, along with the higher VO_2_, indicate a metabolic profile similar to that observed in the CT groups. These results suggest that FMT influences both glucose homeostasis and energy balance, supporting our hypothesis that the nutritional status of the donor host modulates the systemic metabolic effects of the transplanted microbiota.

### Effects of FMT on visceral adipose tissue in recipient mice

**Figure 9.**
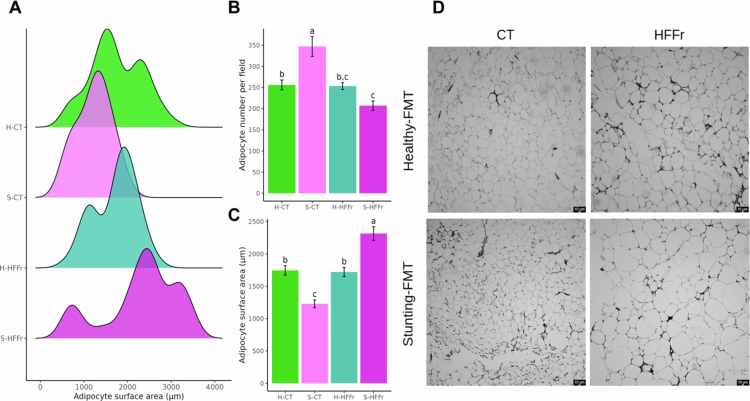
Effects of FMT in the recipient groups on visceral adipose tissue. (A) Distribution of adipocyte subpopulations according to surface area (µm). (B) Adipocyte surface area (µm) (C) Adipocyte number per field. (D) Representative H&E staining of visceral fat depots. Scale bar, 10 μm. The data are expressed as mean ± SEM of at least 5 animals per group. a > b > c > d.

To further investigate how FMT modified adipose tissue morphology, histological analysis of VAT was carried out in FMT recipient mice. The adipocyte size distribution exhibited shifts across the experimental groups according to the frequency of adipocytes with a particular surface area (Figure 9A–D). H-CT mice had a higher frequency of adipocytes measuring 1800 and 2800 µm, S-CT mice had a higher frequency of adipocytes measuring 1500 µm, H-HFFr mice had a higher frequency of adipocytes measuring 1200 and 2000 µm, and H-HFFr mice had a higher frequency of adipocytes measuring 800, 2500, and 3200 µm (Figure 9A). Adipocyte size was also compared among the groups. S-HFFr mice had larger adipocytes than the other groups. No differences were observed when comparing the healthy-FMT groups (H-CT vs. H-HFFr). In contrast, among the mice in the stunting-FMT groups, those in the CT diet resulted in significantly fewer adipocytes (Figure 9B). Moreover, S-CT and S-HFFr mice had the lowest and highest number of adipocytes, respectively, among all experimental groups. The number of adipocytes was significantly greater in the S-HFFr mice than in the groups of CT diet-fed mice. No differences were observed between H-CT and H-HFFr mice (Figure 9C).

VAT hypertrophy was observed in S-HFFr mice. In contrast, hyperplasia and smaller adipocytes were observed in healthy-FMT recipient mice compared to S-HFFr mice. Similarly, S-CT mice exhibited VAT hyperplasia and smaller adipocytes compared to the Healthy-FMT recipient mice. Although VAT hypertrophy was evident in S-HFFr mice, a subgroup of small adipocytes was also present, indicating a limited capacity for VAT expansion. This limitation likely contributes to the insulin resistance and carbohydrate intolerance observed in the stunting-FMT groups.

### Impact on body composition and metabolic phenotype in FMT recipient mice

**Figure 10.**
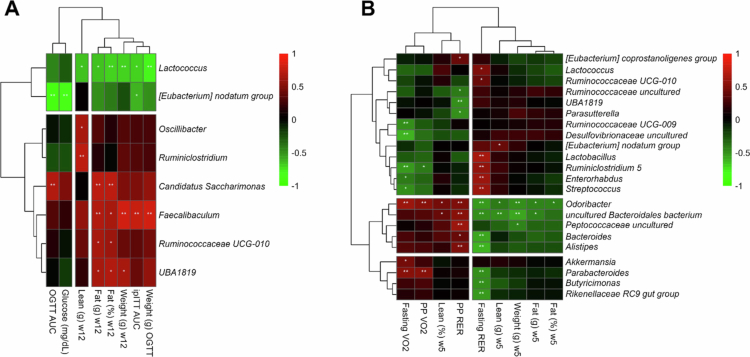
Bacterial genera correlated with biological variables reflecting the metabolic phenotype of recipient mice after FMT. Correlations between the abundances of bacterial genera in the experimental groups and the biological variables bacterial genera with a minimum prevalence of 75% and a minimum relative abundance of 0.1% were retained. (A) Correlation heatmap of bacterial genera at week 5 and variables related to body composition, oxygen consumption and the RER; (B) Correlation heatmap of bacterial genera at week 12 and variables related to body composition insulin resistance and glucose intolerance; (C, D) Log_2_ transformed abundances of correlated bacteria. Significant correlations are indicated as follows: *P* < 0.05 “*”, *P* < 0.001 “**”, and the heatmap clustering method was Euclidean. Red: positive correlations; Green: negative correlations.

We performed a correlation analysis to investigate the association between bacterial genera and phenotypic variables in recipient mice following FMT. The results revealed associations that provide insight into the impact of microbial dynamics on body composition and metabolic phenotypes. Positive correlations with adverse phenotypes were identified for *Lactobacillus, Ruminiclostridium*, *Enterorhabdus*, *Streptococcus*, *Ruminococcaceae uncultured*, *UBA1819*, and *Parasutterella*, linking these taxa to metabolic inflexibility. *The abundances of Candidatus Saccharimonas*, *Faecalibaculum*, *Ruminococcaceae UCG-010*, and *UBA1819* were positively correlated with weight, fat mass, and the AUC for the glucose and insulin tolerance tests. *Lactococcus* and *[Eubacterium] nodatum group* were negatively associated with blood glucose, weight, and fat mass (Figure 10A). In addition, genera associated with a healthy phenotype, including *Odoribacter*, *Bacteroides*, *Alistipes*, *Akkermansia*, *Parabacteroides*, *Butyricimonas*, and *Rikenellaceae_group*, were negatively associated with blood glucose, weight, and fat mass. Furthermore, genera associated with a healthy phenotype, including *Odoribacter*, *Bacteroides*, *Alistipes*, *Akkermansia*, *Parabacteroides*, *Butyricimonas*, and *Rikenellaceae*_*group*, showed negative correlations with body weight, fat mass, and fasting RER and positive correlations with postprandial RER, VO_2_, and lean mass. Notably, the healthy-FMT group was enriched in *Akkermansia* and *Parabacteroides*, which are positively correlated with fasting and postprandial VO_2_, suggesting that healthy-FMT may protect against diet-induced obesity by promoting the growth of taxa associated with increased VO_2_ (Figure 10B). This correlation analysis revealed the complex interplay between bacterial genera and key phenotypic variables, providing valuable insights into the potential mechanisms underlying the impact of FMT on body composition and metabolic health in recipient mice.

### Insights into metagenomic pathways in healthy-FMT and stunting-FMT recipient mice

**Figure 11.**
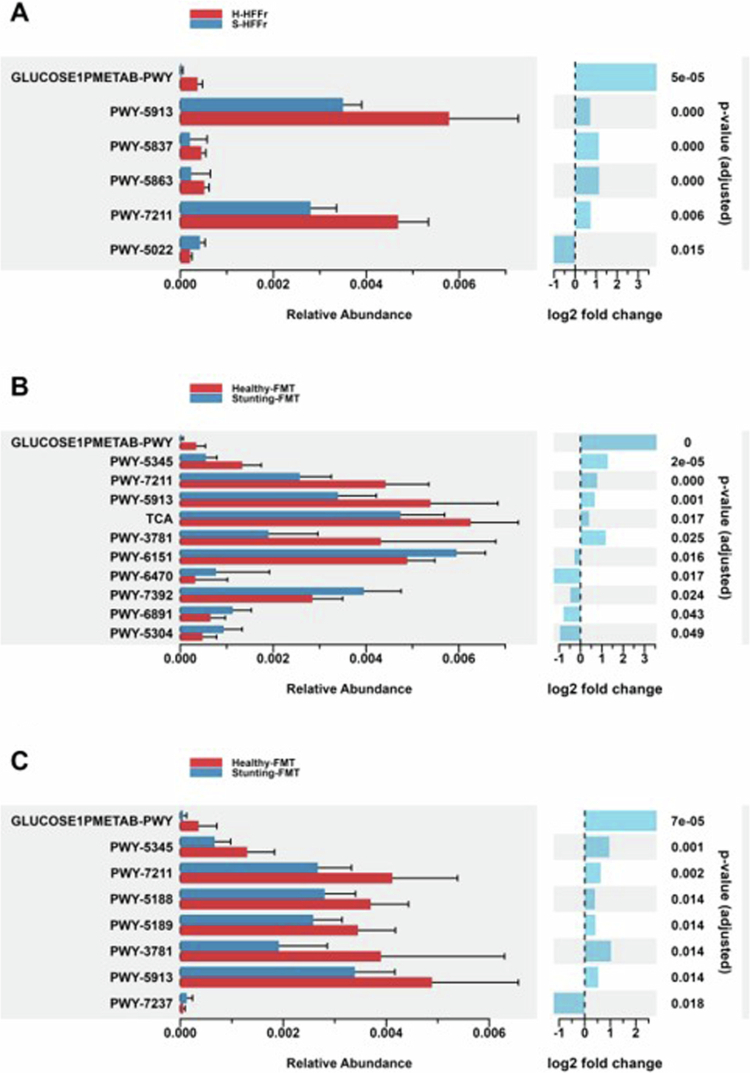
Predicted metabolic pathways by PICRUst2 in FMT recipient mice. (A) Differentially abundant MetaCyc pathways identified at week 5 comparing healthy-FMT and stunting-FMT recipient mice fed with HFFr diet. (B) Differentially abundant pathways identified at week 5 comparing healthy-FMT and stunting-FMT recipient without diet stratification. (C) Global effect in metabolic pathways in healthy-FMT and stunting-FMT recipient mice without diet stratification (fecal samples from weeks 5 and 12 were included).

To determine whether FMT transferred differential functional traits according to the donor’s nutritional status, we compared the predicted metagenomic pathways in recipient mice. At week 5, H-HFFr mice exhibited enrichment in pathways linked to vitamin K biosynthesis, specifically 1, 4-dihydroxy-2-naphthoate (PWY-5837) and phylloquinol biosynthesis (PWY-5863) (Figure 11A–C). Conversely, S-HFFr mice exhibited enrichment in the S-adenosyl-L-methionine cycle I (PWY-6151), peptidoglycan biosynthesis V (*β*-lactam resistance PWY-6470), and sulfur oxidation (PWY-5304) pathways. Notably, the positive regulation of carbohydrate metabolic processes (PWY-5913) was significantly elevated in the H-HFFr group. A broader comparison without time or diet stratification revealed that healthy-FMT mice were enriched in functional capabilities related to glucose metabolism (Glucose1PMETAB_PWY), L-methionine biosynthesis (by sulfhydrylation) (PWY-5345), pyrimidine deoxyribonucleotides de novo biosynthesis (PWY-7211), and biosynthetic pathways of vitamin B12 (PWY-5188 and PWY-5189). Collectively, stunting-FMT recipient mice exhibited reduced functional capabilities associated with glucose metabolism, methionine biosynthesis, and vitamin K and B12 biosynthesis, coupled with an enrichment in *β*-lactam resistance functions. These findings offer valuable insights into the potential impact of the donor’s nutritional status on the transferred functional repertoire.

## Discussion

The present study evaluated whether the gut microbiota of stunted children increases susceptibility to developing obesity later in life upon exposure to an obesogenic diet. The results showed that microbiota from stunted children led to weight gain, glucose intolerance, and insulin resistance in FMT recipient mice fed an HFFr diet. However, FMT from healthy children protects mice against obesity. The approach was based on FMT from stunted children into SPF mice subjected to bowel cleansing. This allowed us to determine whether nutritional status during childhood and exposure to an obesogenic diet could influence the microbiota, potentially promoting the onset of adult obesity. HMA models generally consist of germ-free (GF) or antibiotic-treated mice colonized with human microbiota.^41^^,^^42^ However, GF mice have incomplete maturation of the intestine^43^ and immune system and metabolic abnormalities.^44^^,^^45^ Broad-spectrum antibiotics have several disadvantages, including the outgrowth of antibiotic-resistant bacteria,^46^^,^^47^ loss of body weight,^48^ and direct effects on host tissues, such as reduced gut T-lymphocyte numbers^49^ and thinning of the protective mucus layer due to impaired goblet cell MUC2 production,^50^ a significant concern given the crucial role of mucus in metabolic diseases. Moreover, depending on the initial bacterial diversity, antibiotic concoction, and length of treatment, resistance to antibiotics or residual antibiotics that could persist in recipients and potentially interfere with donor bacteria engraftment may confound the results of FMT research.^51^ Laxative bowel cleansing,^23^ as performed in this study, offers an alternative method to reduce the native gut microbiota before FMT, reducing the bacterial load in mice in a manner comparable to broad-spectrum antibiotics while avoiding the unwanted side effects associated with antibiotic-induced depletion. In our study, we intentionally leveraged the transient disruptive effect of PEG. Bowel cleansing prior to the initial FMT served to reduce the bacterial load and create engraftment niches, and subsequent FMT (without additional PEG treatment) served as a reinforcement. The efficacy of FMT was evidenced by the presence of human-derived ASVs in recipient mice at weeks 5 and 12 alongside baseline ASVs. This observation aligns with previous reports demonstrating the durable coexistence of donor and recipient bacteria after transplantation.^52^ Our findings reveal that, although only a small proportion of the donor microbiota was transferred to recipient mice, it was sufficient to alter their phenotype based on the nutritional status of the donors. This finding aligns with the findings of Llopis et al. that partial microbiota transfer after PEG bowel cleansing can reproduce the phenotypic differences observed in GF models of alcoholic liver disease.^53^ Despite the limitations of partial transfer, our results highlight its biological significance. Hierarchical clustering of the fecal samples demonstrated that, despite the partial transfer of microbiota, FMT consistently shaped the microbial diversity and metabolic phenotype of the recipient mice in a manner dependent on the nutritional status of the fecal donors. Compared to Healthy-FMT, Stunting-FMT promoted greater weight gain in mice fed a HFFr diet than in those fed a CT diet, suggesting that the microbiota from stunted children possesses augmented energy-harvesting capabilities under obesogenic conditions. Although no studies to date have evaluated whether stunting-associated microbial communities increase body weight gain and adiposity when challenged with an obesogenic diet, some have evaluated the transmission of growth phenotypes.^25^ For instance, FMT from 6- and 18-month-old healthy or undernourished Malawian donors into GF mice fed a Malawian diet transmitted growth phenotypes according to the nutritional status of the fecal donor.^54^ A healthy growth phenotype was transferred from healthy Malawian children via the microbial engraftment of growth-discriminatory species, such as *Faecalibacterium prausnitzii*. Conversely, the microbiota of malnourished children is characterized by a loss of growth-supporting taxa and an increase in pathobionts.^54^ Previous studies have focused on determining a causal link between microbiota immaturity in severely stunted or underweight infants and impaired growth. For example, a study in Bangladeshi children confirmed gut microbiota immaturity in severe acute malnutrition (SAM) and moderate acute malnutrition (MAM).^55^ Regarding the microbiota-dependent effects determined by the donor ´ s nutritional status, our results support the hypothesis that early microbial configurations may increase the susceptibility to obesity in adulthood under obesogenic dietary conditions. In this study, stunting-FMT promoted the enrichment of opportunistic pathogens, including *Desulfovibrionaceae*_*uncultured*, *Prevotellaceae*, *Staphylococcus*, and *Helicobacter*^56^^,^^57^, and promoted the engraftment of *Veillonella*, an overrepresented taxon in the small intestine of stunted children from sub-Saharan Africa.^58^ The HFFr diet promoted the enrichment of *Bifidobacterium* only in stunting-FMT recipient mice. Intriguingly, we found no evidence that *Bifidobacterium* ASVs were transferred from fecal donors. Instead, this taxon was present in the recipients at baseline and identified as a PEG-resistant, suggesting that its increased abundance may have been dived indirectly by interactions with taxa introduced through the stunting-FMT. Stunted Bangladeshi children under 38 months have been reported to harbor a greater proportion of *Bifidobacterium longum* than their non-stunted counterparts.^59^ In factorial cross-infection experiments in which phages and bacterial communities were isolated from fresh fecal samples of non-stunted and stunted donors, infection of bacteria from stunted children with phages from non-stunted children resulted in *E. coli* and *B. longum* had the highest species contribution to the *β*-diversity score.^59^ In line with our findings, this phage-mediated modulation may partly explain the enrichment of *Bifidobacterium* in our stunting-FMT recipients despite the absence of direct transfer of *Bifidobacterium* ASVs from donors. This highlights the role of phage-mediated bacterial modulation during FMT, which warrants further investigation. Interestingly, Khan Mirzaei et al. also postulated that higher-than-expected levels of *Bifidobacterium* (*Actinobacteria*) and *Proteobacteria* relative to chronological age may indicate delayed microbiome development or maturity in stunted children.^59^

The enrichment of *Bifidobacterium* may also result from bacterial interactions influenced by increased fructose availability from a HFFr diet. Members of the genus *Bifidobacterium* possess a unique capability to ferment carbohydrates through the fructose-6-phosphate phosphoketolase (F6PPK) pathway.^60^ Microbial dysbiosis induced by high-fructose diets is also known to compromise intestinal barrier integrity, provoke low-grade intestinal inflammation, and cause endotoxemia.^61^ It is plausible that the pathological effects of excessive fructose consumption on barrier function, host metabolism, and inflammation may be mediated, at least in part, by the overgrowth of certain dysbiotic *Bifidobacterium* species.^62^ For example, in mono-colonized mice, the human gut *Bifidobacterium*, including *Bifidobacterium adolescentis*, may induce pro-inflammatory intestinal Th17 cell production.^63^ Furthermore, the human gut microbiota enriched with *Bifidobacterium* has demonstrated the capacity to increase intestinal Th17 cell populations within the small intestines of mice.^64^

Unexpectedly, Healthy-FMT protected mice from weight gain and metabolic complications associated with long-term consumption of an HFFr diet. Among the key taxa linked to this effect was *Akkermansia,* which was enriched in healthy-FMT recipient mice. *Akkermansia muciniphila* is uniquely capable of utilizing mucin as a nutrient source, independent of dietary intake,^65^ which may explain its sustained engraftment under diverse dietary conditions. In addition, our study revealed co-enrichment of *Akkermansia* and *Parabacteroides* in healthy-FMT recipient mice. Although the mutualistic interactions between these taxa remain unclear, their combined presence aligns with metabolic improvements and warrants further mechanistic exploration. For example, *Parabacteroides distasonis* has been implicated in improving metabolic health through pathways involving succinate and secondary bile acid production, which activate intestinal gluconeogenesis and FXR signaling.^66^ These activities are associated with reduced weight gain, improved glucose metabolism, and attenuation of hepatic steatosis. Similarly, reductions in *A. muciniphila* are often reported in obese or overweight children^67^ and correlate with worsened metabolic markers, such as increased BMI, adiposity, and serum glucose and triglyceride levels.^68-70^ In obesity models, *A. muciniphila* and its membrane components, such as the proteins Amuc_1100 and P9, have been shown to modulate lipid metabolism and thermogenesis, likely through the AC3/PKA/HSL ^71^ or AMPK and GLP-1 activation pathways.^72^ These observations suggest that both taxa may contribute to the thermogenic and metabolic benefits observed in healthy-FMT recipients. Mechanistically, it is plausible that *Parabacteroides* and *A. muciniphila* promoted brown adipose tissue thermogenesis *via* succinate dehydrogenase and AMPK activation, respectively. Furthermore, secondary bile acid production by *Parabacteroides* could modulate the intestinal FXR-FGF15 pathway, suppressing hepatic lipogenesis and increasing hepatic fatty acid oxidation, but these pathways were not directly investigated in this study, representing a limitation. Future experiments should focus on quantifying these microbial metabolites and assessing their direct impact on thermogenic and metabolic outcomes and their synergistic effects.

In our study, healthy-FMT in mice fed a HFFr diet induced increased postprandial VO_2_ and increased EE per gram of fat mass. Thus, the adipose tissue of recipient mice was also investigated. VAT analysis further highlighted significant metabolic differences between healthy-FMT and stunting-FMT recipient mice. Healthy-FMT recipient mice fed the HFFr diet exhibited VAT hyperplasia characterized by smaller adipocytes, which are typically associated with improved insulin sensitivity, reduced inflammation, and diminished ectopic lipid accumulation.^73^^,^^74^ In contrast, stunting-FMT resulted in VAT hypertrophy and a reduced number of adipocytes, conditions linked to chronic inflammation and insulin resistance.^75-79^ Importantly, when the subcutaneous adipose tissue reaches its capacity for hyperplasia and hypertrophy, excess fat is routed to the VAT and ectopic sites.^80^ States of extreme hypertrophic obesity are characterized by reduced hyperplasia and further hypertrophic expansion, which accelerate obesity co-morbidities.^81^ Thus, hypertrophic adipocytes are characterized by hypoxia, fibrosis, and chronic inflammatory states, are less responsive to insulin signals, fail to produce adipokines, and have a diminished capacity to store fat. Consequently, the fat “spillover” is directed to other peripheral non-adipose tissues (skeletal muscles, liver, and pancreas), causing cellular malfunction due to the activation of inflammatory and stress response pathways (lipotoxicity) and contributing to the etiology of obesity comorbidity.^78^^,^^79^ Interestingly, the UBA1819 genus was more abundant in the S-HFFr group; this genus was positively correlated with weight and fat mass. Milton-Laskibar demonstrated a higher abundance of the UBA1819 genus in rats with obesity and steatohepatitis induced by a high-fat, high-fructose diet.^82^ In contrast, Pessoa reported that a 60% high-fat diet led to a reduction in the abundance of this genus; however, this effect was no longer evident at 18 weeks, suggesting a potentially transient response.^83^ Furthermore*, the Candidatus Saccharimonas* genus remained enriched until week 12 in the S-HFFr group. This genus was positively associated with weight, fat mass, and the AUC for the glucose tolerance tests. Studies show that *Candidatus Saccharimonas* is more abundant and positively correlated with abnormal lipid metabolism, inguinal white adipose tissue weight, and inflammation, and is negatively correlated with acetic acid and butyric acid in animals fed high-fat and high-fructose diets.^84-86^ Consistent with prior studies, differences observed among S-HFFr and H-HFFr mice highlight that metabolic disturbances, such as adipose hypertrophy and glucose intolerance, can occur despite equivalent caloric intake,^87-90^ emphasizing the role of diet and the microbiota in mediating these effects.

Lipopolysaccharide (LPS) and Toll-like receptor (TLR) ligands also influence the microbiome–adipose axis. Chronic LPS exposure during metabolic endotoxemia compromises gut barrier integrity, amplifies systemic inflammation, and dysregulates adipogenesis via the endocannabinoid (eCB) system. Altered eCB tone, with elevated anandamide and disrupted CB1 receptor signaling, perpetuates adipose dysfunction and gut permeability, establishing a detrimental feedback loop that further disrupts metabolic homeostasis.^91^ Furthermore, *A. muciniphila* noticeably modulates the eCB system tone by increasing the intestinal levels of 2-oleoylglycerol, 2-arachidonoylglycerol, and 2-palmitoylglycerol. Treatment with CB1 antagonists enhances *Akkermansia* abundance, reversing high-fat diet-induced metabolic dysregulation.^92^

These findings emphasize the central role of bacterial metabolites and host signaling pathways in adipose tissue biology. Although our study did not directly measure bacterial metabolites or their systemic effects, the observed phenotypic differences strongly imply their involvement. Future research should prioritize metabolite profiling to validate these mechanisms and clarify how chronic undernutrition shapes microbiota-derived metabolite production and downstream metabolic responses. Understanding these pathways is critical for the development of interventions aimed at preventing obesity and metabolic dysfunction in individuals exposed to undernutrition during childhood. Consistent with prior studies, our results highlight that metabolic disturbances, such as adipose hypertrophy and glucose intolerance, can occur under isocaloric conditions,^87^^,^^89^^,^^90^ emphasizing the role of diet composition and microbiota in mediating these effects.

Our study is the first to demonstrate that the intestinal microbiota from stunted children contributes to the development of obesity, underscoring the importance of microbial composition beyond the first 1000 d of life. The emerging link between childhood undernutrition and adult obesity may explain the increase in obesity in transitioning economies. While most studies focus on children under 5 y of age with SAM or MAM, we highlight the role of the gut microbiota in school-aged children, a population at critical risk during nutritional transitions. Our findings suggest that, beyond the first 1000 d, the gut microbiota of school-aged children plays a critical role in growth and long-term health. Indicators such as the relative microbiota maturity index and the microbiota-for-age z-score (MAZ) have been used to assess microbiota development in children under 2 y of age with SAM. However, developing biomarkers or indicators of gut microbiota maturation in school-aged children could be instrumental in assessing dietary intervention efficacy and identifying individuals at an increased risk of obesity.

This study has certain limitations. While pooling samples from multiple mice provides a broader overview, it inherently lacks the granularity of individual sample analysis. In addition, despite pooled samples, owing to the limited sample size of 2 in the H-HFFr group, a *P*-value cannot be calculated, and observations regarding H-HFFr mice at week 12 were merely descriptive due to the statistical limitations. Furthermore, the use of V_3_–V_4_ 16S rRNA sequencing, although effective to describe the microbiota at the genus level^87^ and useful for our purposes, does not achieve species-level resolution, particularly for closely related taxa such as *Akkermansia*. Future studies employing metagenomic assembly will be critical for fully characterizing the genomes of transferred strains. Moreover, the use of only male mice limits the generalizability of our findings, as sex-specific effects in recipient mice remain unexplored. Although human-to-mouse FMT is a valuable tool for establishing microbiota-driven causal effects, it presents certain limitations, including species-specific differences in immune and metabolic responses. Additionally, murine gastrointestinal anatomy and microbial ecology differ from those of humans, leading to altered microbial stability and function. These constraints mean that FMT models capture only part of the complexity of the human microbiota–host interaction.

Despite these limitations, our findings provide critical insights into the role of the intestinal microbiota in shaping metabolic outcomes, particularly in the context of childhood undernutrition and its long-term implications. Future studies should aim to explore the specific molecular mechanisms and microbial metabolites involved, providing a deeper understanding of their synergistic effects on thermogenic and metabolic pathways.

In conclusion, this is the first study to explore whether the intestinal microbiota of children with stunted growth increases susceptibility to developing obesity in adulthood in an HMA model. Based on our observations, Stunting-FMT recipient mice had increased susceptibility to developing obesity, glucose intolerance, and insulin resistance if an obesogenic environment was favored. In contrast, healthy-FMT conferred protection against diet-induced weight gain and an obesity-like metabolic phenotype by potentially increasing VO_2_ and EE in adipose tissue.

## Supplementary Material

Supplementary Figure 2.jpg Supplementary Figure 2.jpg

Supplementary Figure 1.jpg Supplementary Figure 1.jpg

Supplementary_file_1_ASVs_from_donors_in_FMT_recipients.xlsx Supplementary_file_1_ASVs_from_donors_in_FMT_recipients.xlsx

Supplementary _file_2_Total_ASVs_and_ASVs_by_Diet.xlsx Supplementary _file_2_Total_ASVs_and_ASVs_by_Diet.xlsx

Supplementary Figure 3.jpg Supplementary Figure 3.jpg

Supplementary Figure 4.jpg Supplementary Figure 4.jpg

Supplementary material.docx Supplementary material.docx

## Data Availability

The data presented in this study are available in the article and all the data that support the findings of this study are openly available in NCBI at https://www.ncbi.nlm.nih.gov/bioproject/PRJNA1063615, reference number PRJNA1063615.

## References

[1] Ali MS, Kassahun CW, Wubneh CA, Mekonen EG, Workneh BS. Determinants of undernutrition among private and public primary school children: a comparative cross-sectional study toward nutritional transition in northwest Ethiopia. Nutrition. 2022;96:111575. doi: 10.1016/j.nut.2021.111575. 35077915

[2] Assemie MA, Alamneh AA, Ketema DB, Adem AM, Desita M, Petrucka P, Ambaw MM. High burden of undernutrition among primary school-aged children and its determinant factors in Ethiopia; a systematic review and meta-analysis. Ital J Pediatr. 2020;46:118 Available from: https://pubmed.ncbi.nlm.nih.gov/32847566/. 10.1186/s13052-020-00881-w PMC7448995 32847566

[3] Vaivada T, Akseer N, Akseer S, Somaskandan A, Stefopulos M, Bhutta ZA. Stunting in childhood: an overview of global burden, trends, determinants, and drivers of decline. Am J Clin Nutr [Internet]. 2020;112:777S–791S. doi: 10.1093/ajcn/nqaa159. 32860401 PMC7487433

[4] World Health Organization Nutrition Landscape Information System (‎‎NLiS)‎‎ country profile indicators: interpretation guide. 2019. Geneva: World Health Organization Second edition.

[5] Dewey KG, Begum K. Long-term consequences of stunting in early life. Matern Child Nutr. 2011;7(Suppl 3):5–18 Available from: https://pubmed.ncbi.nlm.nih.gov/21929633/. 21929633 10.1111/j.1740-8709.2011.00349.x PMC6860846

[6] Bentham J, Di Cesare M, Bilano V, Bixby H, Zhou B, Stevens GA, Riley LM, Taddei C, Hajifathalian K, Lu Y, et al. Worldwide trends in body-mass index, underweight, overweight, and obesity from 1975 to 2016: a pooled analysis of 2416 population-based measurement studies in 128·9 million children, adolescents, and adults. Lancet. 2017;390:2627–2642. Available from: https://pubmed.ncbi.nlm.nih.gov/29029897/. 29029897 10.1016/S0140-6736(17)32129-3 PMC5735219

[7] Shama AT, Wakuma O, Debelo S, Terefa DR, Cheme MC, Lema M, Biru B, Geta ET. Prevalence and associated factors of stunting and thinness among primary school-aged children in gudeya bila district, west Ethiopia: a cross-sectional study. BMJ Open. 2023;13:e072313. doi: 10.1136/bmjopen-2023-072313. PMC10201265 37202139

[8] Escher NA, Carrillo-Larco RM, Parnham JC, Curi-Quinto K, Ghosh-Jerath S, Millett C, Seferidi P. Longitudinal transitions of the double burden of overweight and stunting from childhood to early adulthood in India, Peru, and Vietnam. Int J Epidemiol. 2024;53:dyae151. doi: 10.1093/ije/dyae151. 39545485 PMC11565240

[9] Ávila- Curiel A, Juárez-Martínez L, Del Monte-Vega M, Ávila-Arcos M, Galindo-Gómez C, Ambrocio-Hernández R. Estado de Nutrición en Población Escolar Mexicana que Cursa el Nivel de Primaria. 2016. Ciudad de México: DIF Nacional.

[10] Kroker-Lobos MF, Pedroza-Tobias A, Pedraza LS, Rivera JA. The double burden of undernutrition and excess body weight in Mexico. AJCN. 2014;100:1652S–1658S. doi: 10.3945/ajcn.114.083832. 25411308

[11] Shamah-Levy T, Gaona-Pineda EB, Cuevas-Nasu L, Morales-Ruan C, Valenzuela-Bravo DG, Humarán IMG, Ávila-Arcos MA. Prevalencias de sobrepeso y obesidad en población escolar y adolescente de México. ensanut continua 2020-2022. Salud Publica Mex. 2023;65:s218–24. doi: 10.21149/14762. 38060970

[12] 2023) La transición alimentaria en México: una amenaza para la salud humana y planetaria. Available from: https://www.insp.mx/informacion-relevante/la-transicion-alimentaria-en-mexico-una-amenaza-para-la-salud-humana-y-planetaria

[13] Wells JC, Sawaya AL, Wibaek R, Mwangome M, Poullas MS, Yajnik CS, Demaio A. The double burden of malnutrition: aetiological pathways and consequences for health. Lancet. 2020;395:75–88 Available from: https://pubmed.ncbi.nlm.nih.gov/31852605/. 31852605 10.1016/S0140-6736(19)32472-9 PMC7613491

[14] Hoffman DJ, Sawaya AL, Verreschi I, Tucker KL, Roberts SB. Why are nutritionally stunted children at increased risk of obesity? Studies of metabolic rate and fat oxidation in shantytown children from São paulo, Brazil. AJCN. 2000;72:702–707. doi: 10.1093/ajcn/72.3.702. 10966887

[15] Jornayvaz FR, Vollenweider P, Bochud M, Mooser V, Waeber G, Marques-Vidal P. Low birth weight leads to obesity, diabetes and increased leptin levels in adults: the CoLaus study. Cardiovasc Diabetol. 2016;15:73. doi: 10.1186/s12933-016-0389-2. 27141948 PMC4855501

[16] Martin-Gronert MS, Ozanne SE. Metabolic programming of insulin action and secretion. Diabetes Obes Metab. 2012;14:29–39. doi: 10.1111/j.1463-1326.2012.01653.x. 22928562

[17] Grillo LP, Gigante DP, Horta BL, De Barros FCF. Childhood stunting and the metabolic syndrome components in young adults from a Brazilian birth cohort study. Eur J Clin Nutr. 2016;70:548–553. doi: 10.1038/ejcn.2015.220. 26733042 PMC4858756

[18] Murphy EF, Cotter PD, Healy S, Marques TM, O’Sullivan O, Fouhy F, Clarke SF, O’Toole PW, Quigley EM, Stanton C, et al. Composition and energy harvesting capacity of the gut microbiota: relationship to diet, obesity and time in mouse models. Gut. 2010;59:1635–1642. doi: 10.1136/gut.2010.215665. 20926643

[19] Tilg H, Moschen AR, Kaser A. Obesity and the microbiota. Gastroenterology. 2009;136:1476–1483 Available from: https://pubmed.ncbi.nlm.nih.gov/19327360/. 19327360 10.1053/j.gastro.2009.03.030

[20] Zhong W, Lushchak VI. VI. Symphony of digestion: coordinated Host–Microbiome enzymatic interplay in gut ecosystem. Biomolecules. 2025;15:1151. doi: 10.3390/biom15081151. 40867596 PMC12384320

[21] Shapira M. Gut microbiotas and host evolution: scaling up symbiosis. Trends Ecol Evol [Internet]. 2016;31:539–549. doi: 10.1016/j.tree.2016.03.006. 27039196

[22] Méndez-Salazar EO, Ortiz-López MG, Granados-Silvestre M, Palacios-González B, Menjivar M. Altered gut microbiota and compositional changes in firmicutes and proteobacteria in Mexican undernourished and obese children. Front Microbiol. 2018;9:2693. doi: 10.3389/fmicb.2018.02693. 30515137 PMC6251365

[23] Wrzosek L, Ciocan D, Borentain P, Spatz M, Puchois V, Hugot C, Ferrere G, Mayeur C, Perlemuter G, Cassard AM. Transplantation of human microbiota into conventional mice durably reshapes the gut microbiota. NatSR. 2018;8:1–9. doi: 10.1038/s41598-018-25300-3. PMC5931539 29717179

[24] Jalanka J, Salonen A, Salojärvi J, Ritari J, Immonen O, Marciani L, Gowland P, Hoad C, Garsed K, Lam C, et al. Effects of bowel cleansing on the intestinal microbiota. Gut. 2015;64:1562–1568. doi: 10.1136/gutjnl-2014-307240. 25527456

[25] Chibuye M, Mende DR, Spijker R, Simuyandi M, Luchen CC, Bosomprah S, Chilengi R, Schultsz C, Harris VC. Systematic review of associations between gut microbiome composition and stunting in under-five children. NPJ Biofilms Microbiomes. 2024;10:46. doi: 10.1038/s41522-024-00517-5. 38782939 PMC11116508

[26] Turnbaugh PJ, Ley RE, Mahowald MA, Magrini V, Mardis ER, Gordon JI. An obesity-associated gut microbiome with increased capacity for energy harvest. Nature. 2006;444:1027–1031. doi: 10.1038/nature05414. 17183312

[27] WHO Team Nutrition and Food Safety (NFS). WHO child growth standards: growth velocity based on weight, length and head circumference: methods and development. 2009. Available from: https://www.who.int/publications/i/item/9789241547635.

[28] Galarraga M, Campión J, Munõz-Barrutia A, Boqué N, Moreno H, Martínez JA, Milagro F, Ortiz-de-Solórzano C. Adiposoft: automated software for the analysis of White adipose tissue cellularity in histological sections. J Lipid Res. 2012;53:2791–2796. 22993232 10.1194/jlr.D023788 PMC3494244

[29] Thijs S, De Beeck MO, Beckers B, Truyens S, Stevens V, Van Hamme JD, Weyens N, Vangronsveld J. Comparative evaluation of four bacteria-specific primer pairs for 16S rRNA gene surveys. Front Microbiol. 2017;8:494. doi: 10.3389/fmicb.2017.00494. 28400755 PMC5368227

[30] Bolyen E, Rideout JR, Dillon MR, Bokulich NA, Abnet CC, Al-Ghalith GA, Alexander H, Alm EJ, Arumugam M, Asnicar F, et al. Reproducible, interactive, scalable and extensible microbiome data science using QIIME 2. NatBi. 2019;37:852–857. doi: 10.1038/s41587-019-0209-9. PMC7015180 31341288

[31] Callahan BJ, McMurdie PJ, Rosen MJ, Han AW, Johnson AJA, Holmes SP. DADA2: high-resolution sample inference from illumina amplicon data. Nat Methods. 2016;13:581–583. doi: 10.1038/nmeth.3869. 27214047 PMC4927377

[32] McMurdie PJ, Holmes S. Phyloseq: an R package for reproducible interactive analysis and graphics of microbiome census data. PLoS One. 2013;8 e61217. doi: 10.1371/journal.pone.0061217. 23630581 PMC3632530

[33] Segata N, Izard J, Waldron L, Gevers D, Miropolsky L, Garrett WS, Huttenhower C. Metagenomic biomarker discovery and explanation. Genome Biol. 12:1–18. doi: 10.1186/gb-2011-12-6-r60. PMC3218848 21702898

[34] Cao Y, Dong Q, Wang D, Zhang P, Liu Y, Niu C. microbiomeMarker: an R/Bioconductor package for microbiome marker identification and visualization. Bioinformatics. 2022;38:4027–4029. doi: 10.1093/bioinformatics/btac438. 35771644

[35] Dixon P. VEGAN, a package of R functions for community ecology. Journal of Vegetation Science. 2003;14:927–930. doi: 10.1111/j.1654-1103.2003.tb02228.x.

[36] Barnett DJ, Arts ICw, Penders J. microViz: an R package for microbiome data visualization and statistics. J Open Source Softw. 2021;6:3201. doi: 10.21105/joss.03201.

[37] Galili T. Dendextend: an R package for visualizing, adjusting and comparing trees of hierarchical clustering. Bioinformatics. 2015;31:3718–3720. doi: 10.1093/bioinformatics/btv428. 26209431 PMC4817050

[38] Lin H, Eggesbø M, Peddada SD. Linear and nonlinear correlation estimators unveil undescribed taxa interactions in microbiome data. Nat Commun. 2022;13:4946. doi: 10.1038/s41467-022-32243-x. 35999204 PMC9399263

[39] Chong J, Liu P, Zhou G, Xia J. Using MicrobiomeAnalyst for comprehensive statistical, functional, and meta-analysis of microbiome data. Nat Protoc. 2020;15:799–821. doi: 10.1038/s41596-019-0264-1. 31942082

[40] Douglas GM, Maffei VJ, Zaneveld JR, Yurgel SN, Brown JR, Taylor CM, Huttenhower C, Langille MGI. PICRUSt2 for prediction of metagenome functions. NatBi. 2020;38:685–688. doi: 10.1038/s41587-020-0548-6. PMC7365738 32483366

[41] Turnbaugh PJ, Ridaura VK, Faith JJ, Rey FE, Knight R, Gordon JI. The effect of diet on the human gut microbiome: a metagenomic analysis in humanized gnotobiotic mice. Sci Transl Med. 2009;1:6ra14. doi: 10.1126/scitranslmed.3000322. PMC2894525 20368178

[42] Ridaura VK, Faith JJ, Rey FE, Cheng J, Duncan AE, Kau AL, Griffin NW, Lombard V, Henrissat B, Bain JR, et al. Gut microbiota from twins discordant for obesity modulate metabolism in mice. Science (1979). 2013;341:1241214. doi: 10.1126/science.1241214. PMC3829625 24009397

[43] Tomas J, Wrzosek L, Bouznad N, Bouet S, Mayeur C, Noordine ML, Honvo-Houeto E, Langella P, Thomas M, Cherbuy C. Primocolonization is associated with colonic epithelial maturation during conventionalization. FASEB J. 2013;27:645–655. doi: 10.1096/fj.12-216861. 23118025

[44] Gaboriau-Routhiau V, Rakotobe S, Lécuyer E, Mulder I, Lan A, Bridonneau C, Rochet V, Pisi A, De Paepe M, Brandi G, et al. The key role of segmented filamentous bacteria in the coordinated maturation of gut helper T cell responses. Immunity. 2009;31:677–689. doi: 10.1016/j.immuni.2009.08.020. 19833089

[45] Cox LM, Yamanishi S, Sohn J, Alekseyenko AV, Leung JM, Cho I, Kim SG, Li H, Gao Z, Mahana D, et al. Altering the intestinal microbiota during a critical developmental window has lasting metabolic consequences. Cell. 2014;158:705–721. doi: 10.1016/j.cell.2014.05.052. 25126780 PMC4134513

[46] Zhang L, Huang Y, Zhou Y, Buckley T, Wang HH. Antibiotic administration routes significantly influence the levels of antibiotic resistance in gut microbiota. Antimicrob Agents Chemother. 2013;57:3659–3666. doi: 10.1128/AAC.00670-13. 23689712 PMC3719697

[47] Tirelle P, Breton J, Riou G, Déchelotte P, Coëffier M, Ribet D. Comparison of different modes of antibiotic delivery on gut microbiota depletion efficiency and body composition in mouse. BMC Microbiol. 2020;20:1–10. doi: 10.1186/s12866-020-02018-9. 33176677 PMC7657353

[48] Kang JB, Siranosian BA, Moss EL, Banaei N, Andermann TM, Bhatt AS. Intestinal microbiota domination under extreme selective pressures characterized by metagenomic read cloud sequencing and assembly. BMC Bioinform. 2019;20:1–13. doi: 10.1186/s12859-019-3073-1. PMC6886166 31787070

[49] Morgun A, Dzutsev A, Dong X, Greer RL, Sexton DJ, Ravel J, Schuster M, Hsiao W, Matzinger P, Shulzhenko N. Uncovering effects of antibiotics on the host and microbiota using transkingdom gene networks. Gut. 2015;64:1732–1743. doi: 10.1136/gutjnl-2014-308820. 25614621 PMC5166700

[50] Sawaed J, Zelik L, Levin Y, Feeney R, Naama M, Gordon A, Zigdon M, Rubin E, Telpaz S, Modilevsky S, et al. Antibiotics damage the colonic mucus barrier in a microbiota-independent manner. Sci Adv. 2024;10:eadp4119. doi: 10.1126/sciadv.adp4119. 39259805 PMC11389797

[51] Bokoliya SC, Dorsett Y, Panier H, Zhou Y. Procedures for fecal microbiota transplantation in murine microbiome studies. Front Cell Infect Microbiol. 2021;11:711055. doi: 10.3389/fcimb.2021.711055. 34621688 PMC8490673

[52] Li SS, Zhu A, Benes V, Costea PI, Hercog R, Hildebrand F, Huerta-Cepas J, Nieuwdorp M, Salojärvi J, Voigt AY, et al. Durable coexistence of donor and recipient strains after fecal microbiota transplantation. Science. 2016;352:586–589. doi: 10.1126/science.aad8852. 27126044

[53] Llopis M, Cassard AM, Wrzosek L, Boschat L, Bruneau A, Ferrere G, Puchois V, Martin JC, Lepage P, Le Roy T, et al. Intestinal microbiota contributes to individual susceptibility to alcoholic liver disease. Gut. 2016;65:830–839. 26642859 10.1136/gutjnl-2015-310585

[54] Blanton LV, Charbonneau MR, Salih T, Barratt MJ, Venkatesh S, Ilkaveya O, Subramanian S, Manary MJ, Trehan I, Jorgensen JM, et al. Gut bacteria that prevent growth impairments transmitted by microbiota from malnourished children. Science. 2016;351:830 Available from: https://pubmed.ncbi.nlm.nih.gov/26912898/. 10.1126/science.aad3311 PMC4787260 26912898

[55] Subramanian S, Huq S, Yatsunenko T, Haque R, Mahfuz M, Alam MA, Benezra A, Destefano J, Meier MF, Muegge BD, et al. Persistent gut microbiota immaturity in malnourished Bangladeshi children. Nature. 2014;510:417–421. doi: 10.1038/nature13421. 24896187 PMC4189846

[56] Chandra H, Sharma KK, Tuovinen OH, Sun X, Shukla P. Pathobionts: mechanisms of survival, expansion, and interaction with host with a focus on clostridioides difficile. Gut Microbes. 2021;13:1979882. doi: 10.1080/19490976.2021.1979882. 34724858 PMC8565823

[57] Raineri EJM, Maaß S, Wang M, Brushett S, Palma Medina LM, Sampol Escandell N, Altulea D, Raangs E, de Jong A, Vera Murguia E, et al. Staphylococcus aureus populations from the gut and the blood are not distinguished by virulence traits—a critical role of host barrier integrity. Microbiome. 2022;10:1–23. doi: 10.1186/s40168-022-01419-4. 36567349 PMC9791742

[58] Vonaesch P, Morien E, Andrianonimiadana L, Sanke H, Mbecko JR, Huus KE, Naharimanananirina T, Gondje BP, Nigatoloum SN, Vondo SS, et al. Stunted childhood growth is associated with decompartmentalization of the gastrointestinal tract and overgrowth of oropharyngeal taxa. Proc Natl Acad Sci U S A. 2018;115:E8489–98. doi: 10.1073/pnas.1806573115. 30126990 PMC6130352

[59] Khan Mirzaei M, Khan MAA, Ghosh P, Taranu ZE, Taguer M, Ru J, Chowdhury R, Kabir MM, Deng L, Mondal D, et al. Bacteriophages isolated from stunted children can regulate gut bacterial communities in an age-specific manner. Cell Host Microbe. 2020;27:199–212.e5. doi: 10.1016/j.chom.2020.01.004. 32053789 PMC7013830

[60] Anchez BS, Noriega L, Ruas-Madiedo P, Los CG, An R-G, Margolles A. Acquired resistance to bile increases fructose-6-phosphate phosphoketolase activity in bifidobacterium. FEMS Microbiol Lett. 2004;235:35–41. doi: 10.1111/j.1574-6968.2004.tb09564.x. 15158259

[61] Febbraio MA, Karin M. Sweet death”: fructose as a metabolic toxin that targets the gut-liver axis. Cell Metab. 2021;33:2316–2328. doi: 10.1016/j.cmet.2021.09.004. 34619076 PMC8665123

[62] Bootz-Maoz H, Pearl A, Melzer E, Malnick S, Sharon E, Bennet Y, Tsentsarevsky R, Abuchatzera S, Amidror S, Aretz E, et al. Diet-induced modifications to human microbiome reshape colonic homeostasis in irritable bowel syndrome. Cell Rep. 2022;41:111657. doi: 10.1016/j.celrep.2022.111657. 36384106

[63] Tan TG, Sefik E, Geva-Zatorsky N, Kua L, Naskar D, Teng F, Pasman L, Ortiz-Lopez A, Jupp R, Wu HJJ, et al. Identifying species of symbiont bacteria from the human gut that, alone, can induce intestinal Th17 cells in mice. Proc Natl Acad Sci U S A. 2016;113:E8141–50. doi: 10.1073/pnas.1617460113. 27911839 PMC5167147

[64] Ang QY, Alexander M, Newman JC, Tian Y, Cai J, Upadhyay V, Turnbaugh JA, Verdin E, Hall KD, Leibel RL, et al. Ketogenic diets alter the gut microbiome resulting in decreased intestinal Th17 cells. Cell. 2020;181:1263–1275.e16. doi: 10.1016/j.cell.2020.04.027. 32437658 PMC7293577

[65] Derrien M, Vaughan EE, Plugge CM, de Vos WM. Akkermansia municiphila gen. Nov., sp. Nov., a human intestinal mucin-degrading bacterium. Int J Syst Evol Microbiol. 2004;54:1469–1476. doi: 10.1099/ijs.0.02873-0. 15388697

[66] Wang K, Liao M, Zhou N, Bao L, Ma K, Zheng Z, Wang Y, Liu C, Wang W, Wang J, et al. Parabacteroides distasonis alleviates obesity and metabolic dysfunctions via production of succinate and secondary bile acids. Cell Rep. 2019;26:222–235.e5. doi: 10.1016/j.celrep.2018.12.028. 30605678

[67] Pellegrino A, Coppola G, Santopaolo F, Gasbarrini A, Ponziani FR. Role of akkermansia in human diseases: from causation to therapeutic properties. Nutrients. 2023;15:1815. doi: 10.3390/nu15081815. 37111034 PMC10142179

[68] Karcher N, Nigro E, Punčochář M, Blanco-Míguez A, Ciciani M, Manghi P, Zolfo M, Cumbo F, Manara S, Golzato D, et al. Genomic diversity and ecology of human-associated akkermansia species in the gut microbiome revealed by extensive metagenomic assembly. Genome Biol. 2021;22:1–24. doi: 10.1186/s13059-021-02427-7. 34261503 PMC8278651

[69] Schneeberger M, Everard A, Gómez-Valadés AG, Matamoros S, Ramírez S, Delzenne NM, Gomis R, Claret M, Cani PD. Akkermansia muciniphila inversely correlates with the onset of inflammation, altered adipose tissue metabolism and metabolic disorders during obesity in mice. NatSR. 2015;5:1–14. doi: 10.1038/srep16643. PMC4643218 26563823

[70] Parks BW, Nam E, Org E, Kostem E, Norheim F, Hui ST, Pan C, Civelek M, Rau CD, Bennett BJ, et al. Genetic control of obesity and gut microbiota composition in response to high-fat, high-sucrose diet in mice. Cell Metab. 2013;17:141–152. doi: 10.1016/j.cmet.2012.12.007. 23312289 PMC3545283

[71] Zheng X, Huang W, Li Q, Chen Y, Wu L, Dong Y, Huang X, He X, Ou Z, Peng Y. Membrane protein Amuc_1100 derived from akkermansia muciniphila facilitates lipolysis and browning via activating the AC3/PKA/HSL pathway. Microbiol Spectr. 2023;11:e0432322. doi: 10.1128/spectrum.04323-22. 36847500 PMC10100790

[72] Cani PD, Knauf C. A newly identified protein from akkermansia muciniphila stimulates GLP-1 secretion. Cell Metab. 2021;33:1073–1075. doi: 10.1016/j.cmet.2021.05.004. 34077715

[73] Strissel KJ, Stancheva Z, Miyoshi H, Perfield JW, DeFuria J, Jick Z, Greenberg AS, Obin MS. Adipocyte death, adipose tissue remodeling, and obesity complications. Diabetes. 2007;56:2910–2918. doi: 10.2337/db07-0767. 17848624

[74] Nishimura S, Manabe I, Nagasaki M, Seo K, Yamashita H, Hosoya Y, Ohsugi M, Tobe K, Kadowaki T, Nagai R, et al. In vivo imaging in mice reveals local cell dynamics and inflammation in obese adipose tissue. J Clin Invest. 2008;118:710–721 Available from: https://pubmed.ncbi.nlm.nih.gov/18202748/. 18202748 10.1172/JCI33328 PMC2200301

[75] Rajjo TI, Harteneck DA, Jensen MD. Direct free fatty acid storage in different sized adipocytes from the same depot. Obesity (Silver Spring). 2014;22:1275–1279. doi: 10.1002/oby.20673. 24639405 PMC4008637

[76] Boden G. Obesity, insulin resistance and free fatty acids. Curr Opin Endocrinol Diabetes Obes. 2011;18:139–143. doi: 10.1097/MED.0b013e3283444b09. 21297467 PMC3169796

[77] Sakai NS, Taylor SA, Chouhan MD. Obesity, metabolic disease and the pancreas-quantitative imaging of pancreatic fat. Br J Radiol. 2018;91 20180267. 10.1259/bjr.20180267. 29869917 PMC6223168

[78] Symons JD, Abel ED. Lipotoxicity contributes to endothelial dysfunction: a focus on the contribution from ceramide. Rev Endocr Metab Disord. 2013;14:59–68. doi: 10.1007/s11154-012-9235-3. 23292334 PMC4180664

[79] van Herpen NA, Schrauwen-Hinderling VB. Lipid accumulation in non-adipose tissue and lipotoxicity. Physiol Behav. 2008;94:231–241. doi: 10.1016/j.physbeh.2007.11.049. 18222498

[80] Hammarstedt A, Gogg S, Hedjazifar S, Nerstedt A, Smith U. Impaired adipogenesis and dysfunctional adipose tissue in human hypertrophic obesity. Physiol Rev. 2018;98:1911–1941. doi: 10.1152/physrev.00034.2017. 30067159

[81] Jo J, Gavrilova O, Pack S, Jou W, Mullen S, Sumner AE, Cushman SW, Periwal V. Hypertrophy and/or hyperplasia: dynamics of adipose tissue growth. PLoS Comput Biol. 2009;5:e1000324. doi: 10.1371/journal.pcbi.1000324. 19325873 PMC2653640

[82] Milton-Laskibar I, Marcos-Zambrano LJ, Gómez-Zorita S, Carrillo de Santa Pau E, Fernández-Quintela A, Martínez JA, Portillo MP. Involvement of microbiota and short-chain fatty acids on non-alcoholic steatohepatitis when induced by feeding a hypercaloric diet rich in saturated fat and fructose. Gut microbiome (Cambridge, England). 2022;3:e5 Available from: https://pubmed.ncbi.nlm.nih.gov/39295781/. 39295781 10.1017/gmb.2022.2 PMC11406367

[83] Pessoa J, Belew GD, Barroso C, Egas C, Jones JG. The gut microbiome responds progressively to fat and/or sugar-rich diets and is differentially modified by dietary fat and sugar. Nutrients. 2023;15:2097. doi: 10.3390/nu15092097. 37432234 PMC10180990

[84] Yan H, Kuerbanjiang M, Muheyati D, Yang Z, Han J. Wheat bran oil ameliorates high-fat diet-induced obesity in rats with alterations in gut microbiota and liver metabolite profile. Nutr Metab. 2024;21:84. doi: 10.1186/s12986-024-00861-5. PMC11515275 39455992

[85] Zhai Z, Liu J, Niu KM, Lin C, Tu Y, Liu Y, Cai L, Liu H, Ouyang K. Integrated metagenomics and metabolomics to reveal the effects of policosanol on modulating the gut microbiota and lipid metabolism in hyperlipidemic C57BL/6 mice. Front Endocrinol (Lausanne). 2021;12:722055. doi: 10.3389/fendo.2021.722055. 34707567 PMC8542985

[86] Li L, Wang X, Zhou Y, Yan N, Gao H, Sun X, Zhang C. Physalis alkekengi L. Calyx extract alleviates glycolipid metabolic disturbance and inflammation by modulating gut microbiota, fecal metabolites, and glycolipid metabolism gene expression in obese mice. Nutrients. 2023;15(11):2507. doi: 10.3390/nu15112507. 37299470 PMC10255802

[87] Kong CY, Li ZM, Chen HL, Mao YQ, Han B, Guo JJ, Wang LS. An energy-restricted diet including yogurt, fruit, and vegetables alleviates high-fat diet-induced metabolic syndrome in mice by modulating the gut microbiota. J Nutr. 2022;152:2429–2440. doi: 10.1093/jn/nxac181. 36774109

[88] Rendeiro C, Masnik AM, Mun JG, Du K, Clark D, Dilger RN, Dilger AC, Rhodes JS. Fructose decreases physical activity and increases body fat without affecting hippocampal neurogenesis and learning relative to an isocaloric glucose diet. Sci Rep. 2015;5:9589. doi: 10.1038/srep09589. 25892667 PMC4403227

[89] Wang P, Wu T, Fu Q, Liao Q, Li Y, Huang T, Li Y, Zhou L, Song Z. Maternal high-fructose intake activates myogenic program in fetal brown fat and predisposes offspring to diet-induced metabolic dysfunctions in adulthood. Front Nutr. 2022;9:848983 Available from: https://pubmed.ncbi.nlm.nih.gov/35479745/. 35479745 10.3389/fnut.2022.848983 PMC9036479

[90] Pérez B, Torre-Villalvazo I, Wilson-Verdugo M, Lau-Corona D, Muciño-Olmos E, Coutiño-Hernández D, Noriega-López L, Resendis-Antonio O, Valdés VJ, Torres N, et al. Epigenetic reprogramming of H3K4me3 in adipose-derived stem cells by HFS diet consumption leads to a disturbed transcriptomic profile in adipocytes. Am J Physiol Endocrinol Metab. 2024;327:E13–26. doi: 10.1152/ajpendo.00093.2024. 38717362

[91] McLaughlin T, Sherman A, Tsao P, Gonzalez O, Yee G, Lamendola C, Reaven GM, Cushman SW. Enhanced proportion of small adipose cells in insulin-resistant vs insulin-sensitive obese individuals implicates impaired adipogenesis. Diabetologia. 2007;50:1707–1715. doi: 10.1007/s00125-007-0708-y. 17549449

[92] Everard A, Belzer C, Geurts L, Ouwerkerk JP, Druart C, Bindels LB, Guiot Y, Derrien M, Muccioli GG, Delzenne NM, et al. Cross-talk between akkermansia muciniphila and intestinal epithelium controls diet-induced obesity. Proc Natl Acad Sci U S A. 2013;110:9066–9071. doi: 10.1073/pnas.1219451110. 23671105 PMC3670398

