# Supplementary figures and images for: Human fecal transplantation from stunted children promotes metabolic dysfunction in mice fed with a high-fat and high-fructose corn syrup diet
Source: Gut Microbes. 2026 Apr 2;18(1):2651984. doi: 10.1080/19490976.2026.2651984 (PMC13051615; doi:10.1080/19490976.2026.2651984)

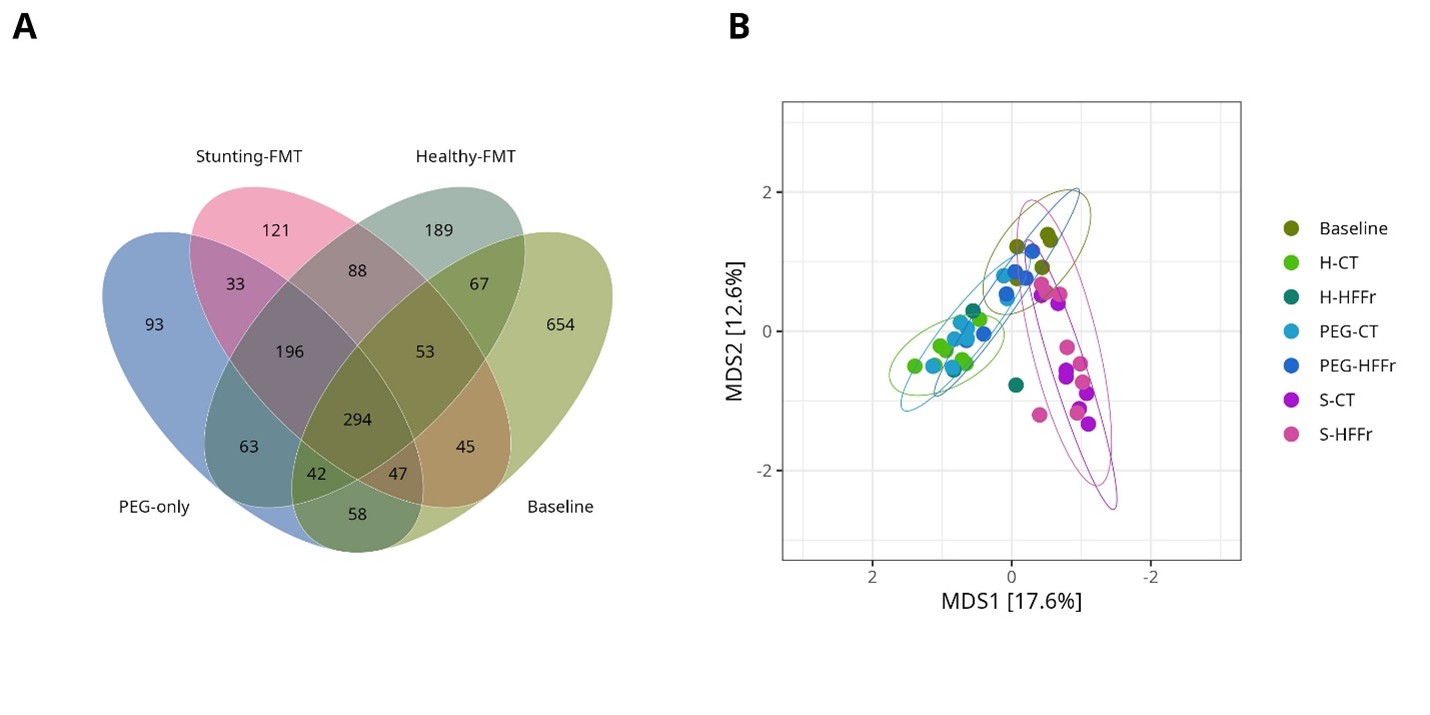

Supplement: Supplementary Figure 2.jpg [file KGMI_A_2651984_SM8046.jpg]

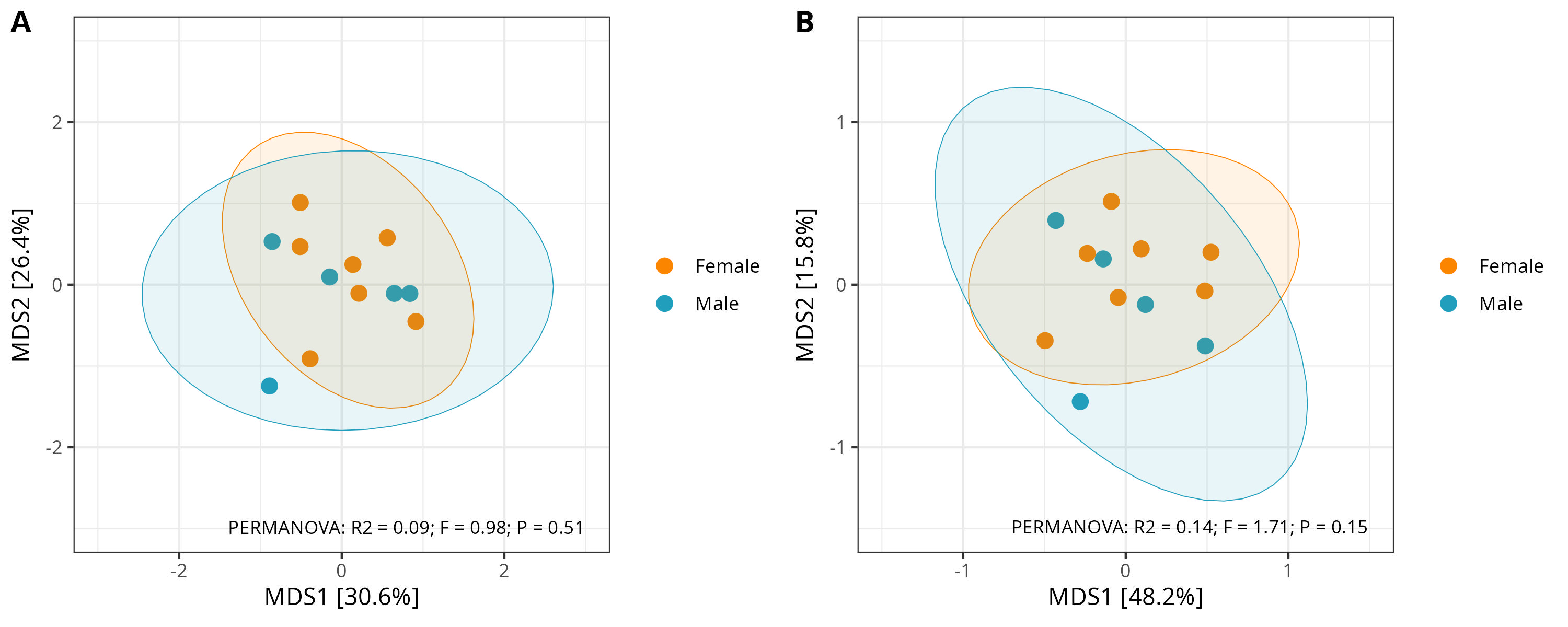

Supplement: Supplementary Figure 1.jpg [file KGMI_A_2651984_SM8041.jpg]

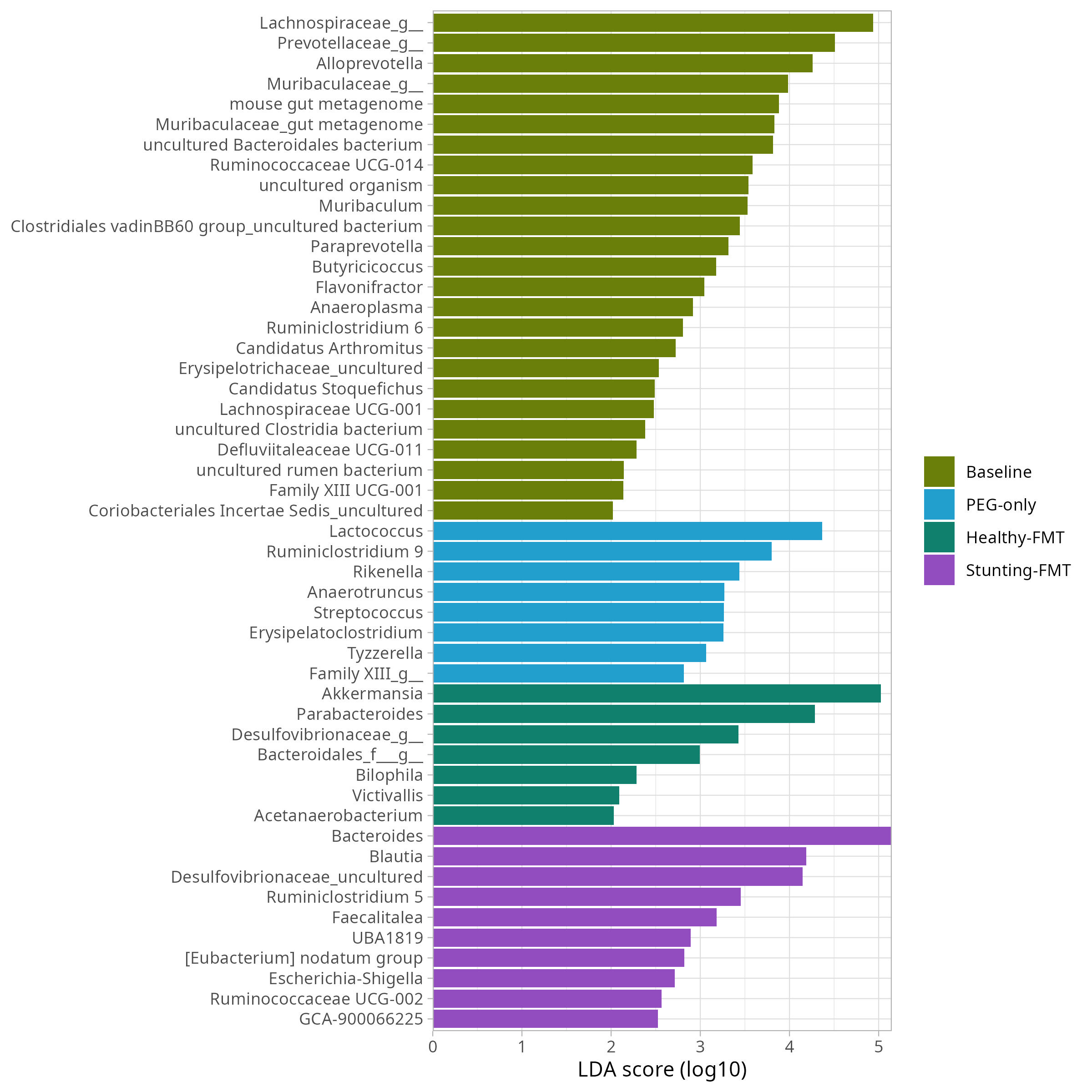

Supplement: Supplementary Figure 3.jpg [file KGMI_A_2651984_SM8044.jpg]

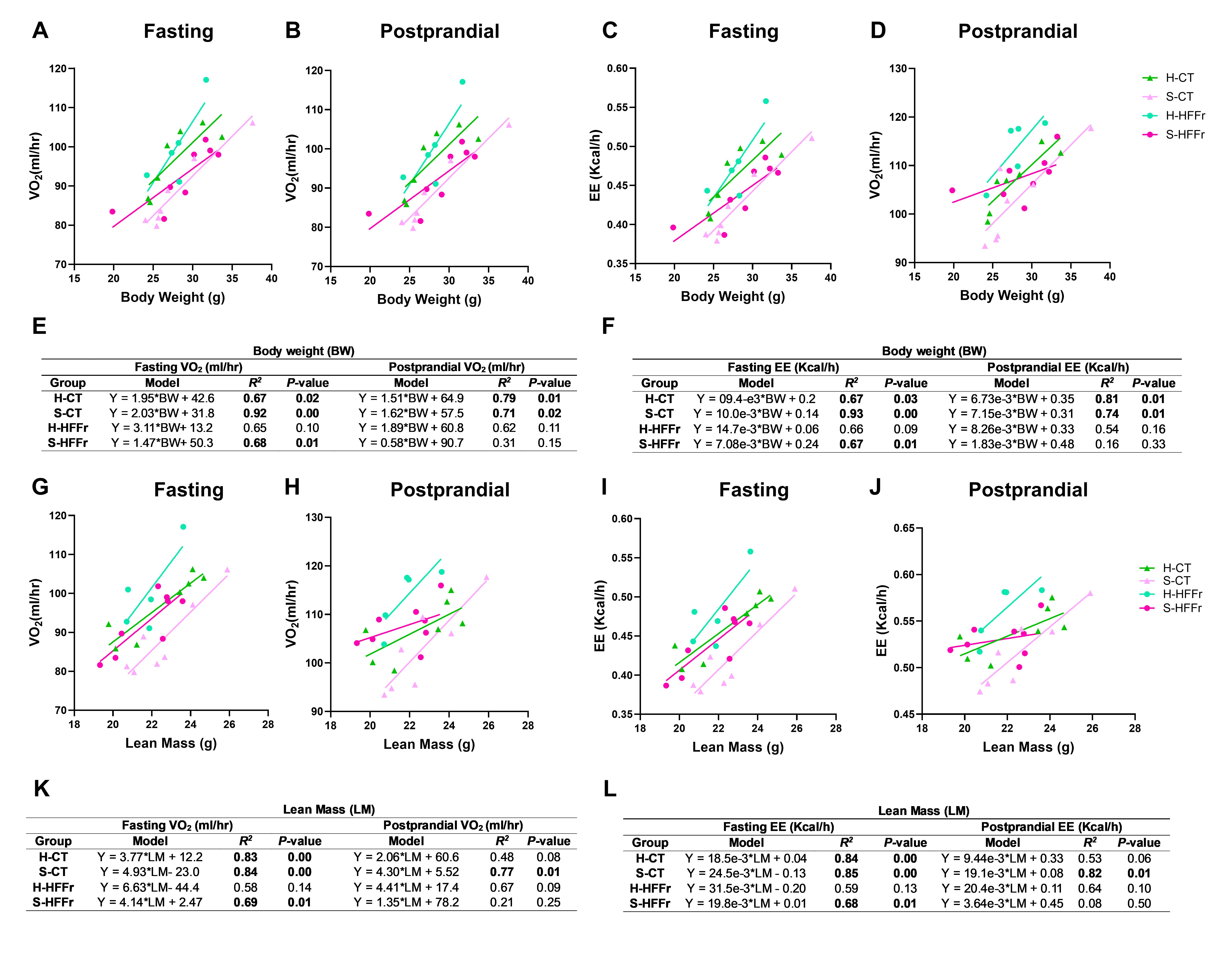

Supplement: Supplementary Figure 4.jpg [file KGMI_A_2651984_SM8045.jpg]
