# Supplementary material for: Human fecal transplantation from stunted children promotes metabolic dysfunction in mice fed with a high-fat and high-fructose corn syrup diet
Source: Gut Microbes. 2026 Apr 2;18(1):2651984. doi: 10.1080/19490976.2026.2651984 (PMC13051615; doi:10.1080/19490976.2026.2651984)
Supplement: Supplementary material.docx [file KGMI_A_2651984_SM1830.docx]

**SUPPLEMENTARY TABLES**

**Supplementary Table 1. Composition of the experimental diets.**

| **Ingredients (g)** | **Control** | **HFFr (15% HCFS-55)** |
| --- | --- | --- |
| **Casein** | 210 | 270 |
| **Starch** | 340 | 300 |
| **Sucrose** | 122 | 10 |
| **Maltodextrin** | 160 | 52 |
| **Soybean oil** | 90 | 100 |
| **Cellulose** | 28 | 18 |
| **AIN-93M-MX mineral mix**  **(Rogers & Harper)** | 35 | 35 |
| **AIN-93-VX vitamin mix**  **(Rogers & Harper)** | 10 | 10 |
| **Choline citrate** | 2 | 2 |
| **Lard** | 0 | 200 |
| **L-Cystine** | 3 | 3 |
| **TBHQ** | 0.0133 | 0.0133 |
| **HFCS-55** | 0 | 15g in 1L of sterilized water *ad libitum.* |

Ingredient composition (per kilogram) of the Control diet and the High-Fat/High-Fructose diet in grams.

**Supplementary Table 2.**

| **Observed Richness** | | | | | | |
| --- | --- | --- | --- | --- | --- | --- |
| **Model** | | **Df** | **Sum of Sq.** | **Mean Sq.** | **F** | **Pr (>F)** |
| **Baseline & FMT recipients (Week 5 post-FMT)** | **Regression** | 6 | 24011 | 4001.9 | 1.02 | 0.43 |
|  | **Residual** | 37 | 145585 | 3934.7 |  |  |
| **Baseline & FMT recipients (Week 12 post-FMT)** | **Regression** | 6 | 50646 | 8441.0 | 2.38 | **0.08** |
|  | **Residual** | 15 | 53209 | 3547.3 |  |  |
| **FMT recipients (Weeks 5 and 12 post-FMT)** | **Regression** | 5 | 8110 | 1621.9 | 0.36 | 0.8745 |
|  | **Residual** | 50 | 226406 | 4528.1 |  |  |

**Supplementary Table 3.**

| **Chao1 index** | | | | | | |
| --- | --- | --- | --- | --- | --- | --- |
| **Model** | | **Df** | **Sum Sq.** | **Mean Sq.** | **F** | **Pr (>F)** |
| **Baseline & FMT-recipients (Week 5 post-FMT)** | **Regression** | 6 | 24,58 | 4,098 | 0.97 | 0.45 |
|  | **Residuals** | 37 | 156,31 | 4,224 |  |  |
| **Baseline & FMT-recipients (Week 12 post-FMT)** | **Regression** | 6 | 50,394 | 8,398 | 2.35 | 0.083 |
|  | **Residuals** | 15 | 53,531 | 3,568 |  | |
| **FMT-recipients (Weeks 5 and 12 post-FMT)** | **Regression** | 11 | 57,142 | 5,194 | 1.201 | 0.314 |
|  | **Residuals** | 44 | 190,248 | 4,323 |  | |

**Supplementary Table 4.**

| **Simpson Index** | | |  |
| --- | --- | --- | --- |
|  | **χ² (chi-squared)** | **Df** | **p-value** |
| **Baseline & FMT recipients (Week 5 post-FMT)** | 9.6348 | 6 | 0.1409 |
| **Baseline & FMT recipients (Week 12 post-FMT)** | 5.6964 | 6 | 0.4580 |
| **Baseline & FMT recipients (Week 12 post-FMT)** | 11.2310 | 11 | 0.4242 |

**Supplementary Table 5. Bray-Curtis beta diversity of experimental groups at week 5**

| **Factor** | **Df\|** | **Sum of Squares** | **R²** | ***F*-value** | ***P*-value** |
| --- | --- | --- | --- | --- | --- |
| **Donor** | 2 | 2.79 | 0.34 | 7.32 | **0.001** |
| **Diet** | 1 | 0.39 | 0.04 | 2.06 | **0.023** |
| **Group** | 1 | 0.22 | 0.02 | 1.17 | 0.25 |
| **Residual** | 25 | 4.77 | 0.58 |  |  |
| **Total** | 29 | 8.18 | 1 |  |  |

PERMANOVA of Bray-Curtis beta diversity among FMT-recipient mice at week 5. Permutation test under reduced model (999 permutations).

**Supplementary Table 6. Bray-Curtis beta diversity of experimental groups at week 12**

| **Factor** | **Df** | **Sum of Squares** | **R²** | ***F*-value** | ***P*-value** |
| --- | --- | --- | --- | --- | --- |
| **Week** | 1 | 0.57 | 0.06 | 2.99 | **0.001** |
| **Donor** | 1 | 1.50 | 0.17 | 7.87 | **0.001** |
| **Diet** | 1 | 0.52 | 0.05 | 2.73 | **0.005** |
| **Group** | 1 | 0.26 | 0.03 | 1.39 | 0.12 |
| **Residual** | 31 | 5.92 | 0.67 | — | — |
| **Total** | 35 | 8.78 | 1 | — | — |

PERMANOVA of Bray-Curtis beta diversity among FMT-recipient mice at weeks 5 and 12. Permutation test under reduced model (999 permutations).

**Supplementary Table 7. Bray-Curtis beta diversity of experimental groups and PEG-treated mice at week 5**

| **Factor** | **Df** | **Sum of Squares** | **R²** | ***F*-value** | ***P*-value** |
| --- | --- | --- | --- | --- | --- |
| **Donor** | 3 | 3.61 | 0.30 | 6.36 | **0.001** |
| **Diet** | 1 | 0.58 | 0.04 | 3.09 | **0.001** |
| **Group** | 2 | 0.65 | 0.05 | 1.72 | **0.010** |
| **Residual** | 37 | 7 | 0.59 |  |  |
| **Total** | 43 | 11.85 | 1 |  |  |

PERMANOVA of Bray-Curtis beta diversity of FMT-recipient mice and PEG-treated groups at week 5. Permutation test under reduced model (999 permutations).

**Supplementary Table 8. Effect of PEG-cleansing and FMT in microbiota diversity in Healthy-FMT mice.**

| **Factor** | **Df** | **Sum of Squares** | **R^2^** | ***F*-Value** | ***P*-value** |
| --- | --- | --- | --- | --- | --- |
| **Healthy-FMT** | 1 | 0.82 | 0.13 | 4.16 | **0.001** |
| **PEG-Cleansing** | 1 | 1.20 | 0.18 | 6.10 | **0.001** |
| **Residual** | 23 | 4.53 | 0.69 |  |  |
| **Total** | 55 | 6.55 | 1.0 |  |  |

PERMANOVA of Bray-Curtis beta diversity of Baseline, PEG-treated and Healthy-FMT mice at week 5. Permutation test under reduced model (999 permutations).

**Supplementary Table 9. Effect of PEG-cleansing and FMT in microbiota diversity in Stunting-FMT mice.**

| **Factor** | **Df** | **Sum of Squares** | **R^2^** | ***F*-Value** | ***P*-value** |
| --- | --- | --- | --- | --- | --- |
| **Stunting-FMT** | 1 | 1.35 | 0.14 | 6.24 | **0.001** |
| **PEG-Cleansing** | 1 | 1.20 | 0.12 | 5.56 | **0.001** |
| **Residual** | 30 | 6.70 | 0.72 |  |  |
| **Total** | 33 | 9.26 | 1.0 |  |  |

PERMANOVA of Bray-Curtis beta diversity of Baseline, PEG-treated and Stunting-FMT mice at week 5. Permutation test under reduced model (999 permutations).

**SUPPLEMENTARY FIGURES**

**Supplementary Figure 1. Bacterial diversity in FMT donors stratified by sex. A)** Bray-Curtis-based and **B)** weighted Unifrac-based principal coordinate analysis (PCoA) of fecal donors by sex.

**Supplementary Figure 2. Influence of PEG in bacterial engraftment and diversity in FMT-recipient mice. A)** Venn diagram of ASVs shared between I-Healthy and I-Stunting fecal inocula, mice at Baseline and post-FMT (Stunting-FMT and Healthy-FMT without time stratification). **B)** Bray-Curtis-based principal coordinate analysis (PCoA) of PEG-only treated mice and FMT-recipient mice in Baseline and post-FMT at week 5.

**Supplementary Figure 3. Comparison of PEG-cleansing and FMT effects in taxon enrichment in FMT recipient mice.** Linear Discriminant Analysis of Effect Size (LDA-LefSe) at the genus level comparing Baseline, PEG-only, Healthy-FMT and Stunting-FMT groups. LDA score threshold <0.2 and *P*-value <0.05 was considered statistically significant.

**Supplementary Figure 4. Linear regression analysis of energy expenditure and oxygen consumption in FMT-recipient mice.** Effect of FMT in; **A-B)** oxygen consumption and **C-D)** energy expenditure in mice according to body weight. **E-F)** Linear regression equations for each group according to body weight in grams. Effect of FMT in; **G-H)** oxygen consumption and **I-J)** energy expenditure in mice according to lean mass in grams. **K-L)** Linear regression equations for each group according to lean mass in grams. (n = 5–8 by group).
